# mRNA vaccines in disease prevention and treatment

**DOI:** 10.1038/s41392-023-01579-1

**Published:** 2023-09-20

**Authors:** Gang Zhang, Tianyu Tang, Yinfeng Chen, Xing Huang, Tingbo Liang

**Affiliations:** 1 Zhejiang Provincial Key Laboratory of Pancreatic Disease, The First Affiliated Hospital, Zhejiang University School of Medicine, 310009 Hangzhou, Zhejiang China; 2 Department of Hepatobiliary and Pancreatic Surgery, The First Affiliated Hospital, Zhejiang University School of Medicine, 310003 Hangzhou, Zhejiang China; 3 Zhejiang Clinical Research Center of Hepatobiliary and Pancreatic Diseases, 310003 Hangzhou, Zhejiang China; 4 The Innovation Center for the Study of Pancreatic Diseases of Zhejiang Province, 310009 Hangzhou, Zhejiang China; 5 Cancer Center, Zhejiang University, 310058 Hangzhou, Zhejiang China

**Keywords:** Vaccines, Immunological techniques

## Abstract

mRNA vaccines have emerged as highly effective strategies in the prophylaxis and treatment of diseases, thanks largely although not totally to their extraordinary performance in recent years against the worldwide plague COVID-19. The huge superiority of mRNA vaccines regarding their efficacy, safety, and large-scale manufacture encourages pharmaceutical industries and biotechnology companies to expand their application to a diverse array of diseases, despite the nonnegligible problems in design, fabrication, and mode of administration. This review delves into the technical underpinnings of mRNA vaccines, covering mRNA design, synthesis, delivery, and adjuvant technologies. Moreover, this review presents a systematic retrospective analysis in a logical and well-organized manner, shedding light on representative mRNA vaccines employed in various diseases. The scope extends across infectious diseases, cancers, immunological diseases, tissue damages, and rare diseases, showcasing the versatility and potential of mRNA vaccines in diverse therapeutic areas. Furthermore, this review engages in a prospective discussion regarding the current challenge and potential direction for the advancement and utilization of mRNA vaccines. Overall, this comprehensive review serves as a valuable resource for researchers, clinicians, and industry professionals, providing a comprehensive understanding of the technical aspects, historical context, and future prospects of mRNA vaccines in the fight against various diseases.

## Introduction

Vaccines have proven remarkable efficacy in preventing the spread of infectious diseases, causing the preservation of countless lives annually.^1,2^ The extensive implementation of vaccines in recent decades has led to the elimination of smallpox and an extremely low incidence of polio, measles, and other infectious diseases.^3^ The World Health Organization reports that vaccination prevents approximately 2 million mortalities from measles, influenza, pertussis, and tetanus every year.^4^ Moreover, the use of vaccines in cancer management shows potent efficacy in preclinical trials, becoming one of the most promising treatments in the field of immune oncology and gaining more attention than ever. Conventional vaccines have several disadvantages that may limit their application in disease prevention and treatment. For instance, the underlying procedure for the development of dendritic cell (DC) vaccines involves a labor-intensive and time-consuming process that necessitates the preparation of patient-autologous cells. The engineering and fabrication of microorganism-based vaccines entail intricate and complex processes. Peptide vaccines exhibit MHC restriction, selectively activating monoclonal T cells, thus having a high risk of immune escape.^5^ DNA vaccines have risks of genomic alteration, long-term expression, and generation of anti-DNA autoantibodies that might impede their utilization in humans.^6,7^ Therefore, it is essential to select a suitable vaccine format with promising value for disease prevention and treatment.

**Fig. 1:**
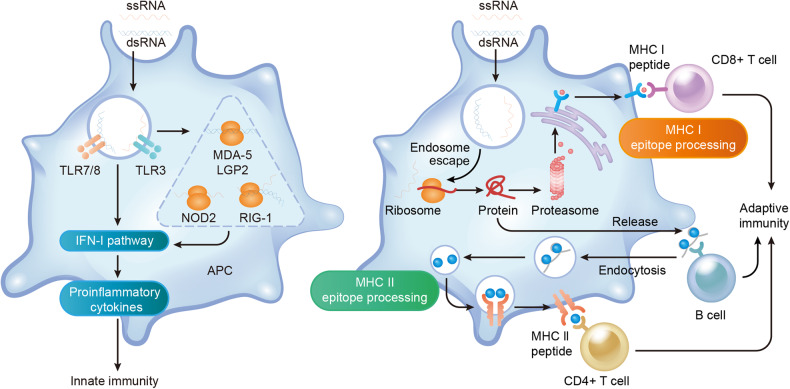
Dual effects of mRNA vaccine on immune activation. mRNA vaccines induce both innate and adaptive immunity. Endocytosis of exogeneous mRNA by antigen presenting cells is sensed by TLR3 and TLR7/8 in the endosomes as well as RIG-1, NOD2, LGP2, and MDA-5 in the cytosol, inducing strong IFN-I responses, then triggering proinflammatory cytokine production, thereby activating innate immunity (left). mRNA-encoded protein is released out of the cell to activate B cells, while mRNA-encoded or re-endocytosed proteins are degraded as peptides in the proteasome to be presented on MHC-I or MHC-II molecules to activate CD4^+^ and CD8^+^ T cells, cocontributing to adaptive immunity activation (right). This figure is created using Adobe Illustrator and is inspired by these two papers^257,258^

Vaccines using messenger RNA (mRNA), a single nucleotide sequence that functions as a template for protein translation, possess multiple beneficial features over traditional vaccines.^3,8^ Indeed, mRNA vaccines use the body cell as the core facility for a natural induction of both innate and adaptive immunity (Fig. 1), enabling posttranslational modification and full functionality of protein products, allowing the correct translation folding and assembly in the host cells of multimeric and versatile proteins that cannot be produced in bioreactors, and allowing the transfer of the produced intracellular and transmembrane proteins to their suitable cellular locations.^9–14^ mRNA vaccines can be designed to encode any antigen based on the unique attributes of diseases. Moreover, compared with DNA vaccines, mRNA vaccines avoid the potential risk of insertional mutagenesis in the host genome and cause adjustable expression of the selected antigen.^15–17^ From the commercial point of view, the mRNA vaccine allows rapid development and large-scale production through a cell-free process due to the highly productive transcription reaction in vitro, which is also extremely cost-effective.^1,3,7,15–17^ Notably, although mRNA itself also has several disadvantages compared with other vaccine modalities (e.g., poor stability and potent immunogenicity that limit the usage of mRNA vaccines in vivo), improvements in modifications and delivery largely address these obstacles, ensuring the maintenance of in vivo stability as well as the balance between the initiation of a robust immune responses and irreversible adverse reactions caused by lasting function.^1,3,7,12,15–20^ For all these reasons, mRNA vaccines have emerged as a promising modality in the prevention and treatment of a number of diseases.

This comprehensive review provides an in-depth exploration of the technical foundations of mRNA vaccine, encompassing essential aspects such as mRNA design, synthesis, delivery, and adjuvant technologies. This comprehensive review presents a methodical and structured analysis of representative mRNA vaccines used in a diverse array of medical conditions, including infectious diseases, cancers, immunological diseases, tissue damages, and rare diseases. Furthermore, this review includes a forwards-looking discourse on the current obstacles and potential possibilities in the development and implementation of mRNA vaccines.

## mRNA vaccine development

**Fig. 2:**
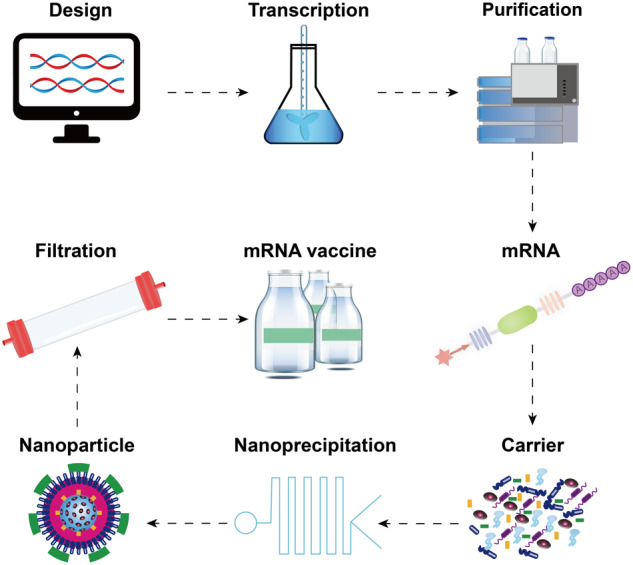
Pipeline for the development of mRNA vaccines. The development of mRNA vaccines includes a series of steps, including sequencing design, in vitro transcription, purification, nanoprecipitation, and filtration. This figure is created using Adobe Illustrator and refers to this paper^4^

The development of mRNA vaccines is the culmination of extensive research spanning several decades. The discovery of mRNA dates back to 1961, and its isolation for in vitro protein expression was first achieved in 1969.^21,22^ In 1990, in vitro transcribed mRNA was successfully validated as a template for synthesizing proteins in mouse skeletal muscle cells in vivo, marking a breakthrough in in vivo mRNA expression and laying the groundwork for the development of mRNA vaccines.^23^ In 1992, vasopressin mRNA was injected into the hypothalamus, successfully expressed, and yielded physiological responses.^24^ Subsequently, in 1993 and 1995, mRNA was found to elicit both innate and adaptive immunity.^25–27^ Despite these promising findings, the development of mRNA vaccines initially faced limited investment, mainly owing to concerns over their instability, inefficient in vivo transportation, and possible innate immunogenicity. However, due to their safety, straightforward design, and simplicity of manufacturing, research on mRNA has persevered. Ultimately, this persistence paid off, as evidenced by the development of highly effective mRNA vaccines against COVID-19, which have played a pivotal role in the ongoing efforts to control the pandemic. To date, a comprehensive framework has been established for the development of mRNA vaccines, including design, synthesis, and delivery technologies (Fig. 2).

### mRNA design

The advancement of mRNA vaccines faced a major obstacle owing to the instability of mRNAs and poor translational efficiencies.^4,28^ In vitro, transcribed mRNA comprises five primary elements, namely, the 5ʹ cap, 5ʹ untranslated region (UTR), an open reading frame (ORF), 3ʹ UTR, and a poly(A) tail, all of which simulate the structure of an endogenous mRNA.^4,28^ To enhance mRNA translational efficacy, scientists have devised various techniques to modify each of these components and optimize mRNA design.

The 5′ cap, a modified nucleotide structure situated at the 5′ end of the mature mRNA molecule, comprises a guanine nucleotide linked to the mRNA via a triphosphate linkage, with additional methylations at the 7th position of the guanine and/or the 2′ position of the first transcribed nucleotide.^29,30^ It has vital roles in several aspects of mRNA stability and functions, including protection against exonucleases, enhancement of mRNA translation efficiency, and facilitation of transport from the nucleus to the cytoplasm.^4,31^ In mRNA vaccines, the inclusion of a 5′ cap structure is critical for the stabilization of mRNA molecules and the promotion of efficient translation to the encoded protein. Notably, the 5′ cap modification, particularly the m7G cap, boosts mRNA translational efficiency by facilitating its recognition by the translation initiation complex.^4,32^ Furthermore, prior researches have highlighted the capability of the m7G cap to protect mRNA from nucleases, thereby enhancing its stability and immunogenicity.

The poly(A) tail is a critical posttranscriptional modification of mRNA that significantly contributes to its stability, export, and translation. In eukaryotic cells, the process of mRNA maturation involves the addition of a long chain of adenine nucleotides at the 3′ end of the mRNA molecule, with a typical length ranging from 50–250 nucleotides.^31,33^ A key function of the poly(A) tail is to safeguard mRNA from exonucleases, which are enzymes that can degrade RNA from its ends.^29,31^ Additionally, the poly(A) tail facilitates the export of mRNA from the nucleus to the cytoplasm, wherein it can be translated into proteins.^34–36^ Furthermore, the poly(A) tail is involved in the initiation of protein synthesis.^34–36^ It interacts with poly(A)-binding protein, which recruits the ribosome to the mRNA, thus promoting efficient translation.^34–36^ The introduction of the poly(A) tail in mRNA vaccines serves two critical purposes. First, it stabilizes the mRNA molecule and protects it from degradation by cellular enzymes. Second, it enhances the mRNA’s translation efficiency, leading to increased expression of the antigen and a more potent immune response. The length of the poly(A) tail in mRNA vaccines is meticulously optimized to balance mRNA stability and translation efficiency.

The UTRs of mRNA play a crucial role in the regulation of gene expression.^31,32^ Located at the 5′ and 3′ ends, these regions are involved in the control of mRNA stability, translation efficiency, and subcellular localization, thereby regulating the production and function of the corresponding protein.^4^ The coding sequence of mRNA determines the protein sequence, while the UTRs regulate its expression. Specifically, the 5′ UTR plays critical roles in regulating mRNA stability and translational efficiency, with modifications to the 5′ cap structure and length of the 5′ UTR enhancing the two.^28,37^ Alternative splicing of the 5′ UTR can alter the translational efficiency of mRNA.^37^ Similarly, the 3′ UTR regulates mRNA stability through the binding of regulatory proteins and microRNAs, which can either destabilize or stabilize mRNA. Modifying the 3′ UTR, for instance, by adding poly(A) tails, can enhance mRNA stability and protein expression. In mRNA vaccine design, UTRs are meticulously engineered to optimize protein expression and immune responses.^29^ The 5′ UTR can be modified to enhance translation efficiency, while the 3′ UTR can be modified to stabilize mRNA and prolong protein expression, resulting in improved immunogenicity and efficacy of mRNA vaccines.^37^

The ORF, beginning with a start codon and ending with a stop codon, is a critical segment of mRNA translated into a protein by the ribosome.^4,28^ The length of the ORF can vary from a few hundred to several thousand nucleotides.^38,39^ The sequence of the ORF is responsible for determining the identity and structure of the protein synthesized, thus playing a pivotal role in the effectiveness of mRNA.^38,39^ In the context of mRNA vaccines, the importance of ORF design is paramount, as it directly affects the production of the target antigen. Advances in mRNA vaccine technology have facilitated their rapid design and production against emerging infectious diseases. ORF sequences have been optimized to enhance mRNA stability and translation efficiency. One such approach involves optimizing the codon usage of the ORF, thereby improving translation efficiency and reducing premature termination.^4,28,40^ Another strategy involves incorporating specific RNA modifications, including a pseudouridine, to enhance the stability and accuracy of mRNA translation.^4,16,28,41^ Additionally, the use of nonnatural amino acids in the ORF can expand the epitope repertoire presented by the antigen, thereby potentially inducing a broader immune response. The development of efficient and effective ORF design strategies is vital for the success of mRNA vaccines. These endeavors are expected to result in the development of more potent and versatile mRNA vaccines with broad application prospects for disease prevention and treatment.

Notably, modified nucleosides have gained widespread popularity within mRNA technology owing to their ability to enhance the stability, translational efficiency, and immunogenicity of mRNA molecules.^42–44^ These nucleoside analogs can be integrated into the mRNA sequence in in vitro transcription, resulting in the formation of modified mRNA molecules with superior properties relative to their unmodified counterparts. Among the most frequently employed modified nucleosides in mRNA are pseudouridine, 5-methylcytidine, and 2-thiouridine.^42–45^ Pseudouridine improves mRNA stability and translational efficiency while reducing the activation of innate immune responses.^45^ 5-Methylcytidine elevates protein expression levels, while 2-thiouridine enhances the precision of translation by increasing the binding affinity between mRNA and ribosomes.^45^ Other modifications, including N1-methylpseudouridine and 5-methoxyuridine, have also been utilized to improve mRNA stability and translation efficiency.^45^ The incorporation of modified nucleosides in mRNA technology holds considerable promise for the development of more effective and safer mRNA-based therapeutics, including vaccines and gene therapies for a wide range of human diseases.

### mRNA vaccine synthesis

The production of mRNA by in vitro transcription involves the use of RNA polymerase enzymes for synthesizing mRNA from a DNA template outside a living cell. The upstream process entails using a plasmid as a template and transcribing it into primary mRNA using T7, SP6, or T3 RNA polymerase.^3^ This reaction takes only a few hours and yields a few milligrams of primary mRNA per milliliter of reaction. Subsequently, capping of primary mRNA occurs during transcription using a Cap analog instead of the natural substrate or via a two-step enzymatic reaction using RNA 2′-O-ribose transferase, RNA methyltransferase, and a methyl donor substrate.^46–49^ Although utilizing Cap analogs is a rapid and practical approach to cap mRNA, its employment is impeded by the relatively high costs and the instability associated with the resultant m7GpppN cap structure.^4,49^ Conversely, the two-step enzymatic reaction produces a more authentic and stable m7GpppN cap structure, albeit requiring additional steps and enzyme reactions, as well as a meticulous selection of suitable enzymes and methyl donor substrates.^28^ To meet clinical quality standards, the mRNA generated upstream needs to undergo multiple purification steps to separate and purify it from the reaction mixture. Size exclusion chromatography is a commonly utilized method for separating mRNA molecules based on their sizes and shapes.^50–52^ This approach is both simple and gentle, making it effective for removing impurities, including residual DNA, RNA, and proteins. Reverse-phase high-performance liquid chromatography separates mRNA molecules based on their hydrophobicity, thus providing high resolution and purity, but it can be time-consuming and requires expensive equipment. Affinity chromatography is another strategy for purifying mRNA vaccines, whereby specific ligands are used to capture and purify the mRNA molecules.^32,50,51^ This method can provide high specificity and yield but may require additional steps for ligand immobilization and can be costly. Ion exchange chromatography is another common method for mRNA purification, which separates molecules based on their charge.^29,50,51^ Although this method has high yield and purity, it may require multiple steps and careful optimization to achieve optimal results. In addition to chromatography-based methods, precipitation-based approaches, including isopropanol or ethanol precipitation, can also be used to purify mRNA vaccines.^51^ These methods are simple and cost-effective but are less effective in removing impurities, and additional steps for resuspension and quality control may be needed. Ultimately, the purification method chosen for mRNA vaccines depends on various factors, including the desired purity level, scalability, cost, and downstream applications.

### mRNA vaccine delivery

The delivery of mRNA vaccines into cells presents significant challenges due to the inherent instability of RNA and the need to protect it from degradation in the extracellular environment. Over the past few decades, researchers have explored various delivery systems to overcome these challenges and enhance the efficacy of mRNA vaccines.

One of the earliest approaches was the use of naked mRNA molecules, which were directly injected into cells or tissues.^4,28,30^ Herein, mRNA is delivered without a carrier, allowing it to be translated into antigen proteins within cells. While naked mRNA vaccines are relatively easy to produce and have shown promise in preclinical studies, they are less stable and may elicit weaker immune responses than mRNA vaccines delivered with carriers.^38^ Another early approach was the mRNA-DC vaccine, which involved the loading of DCs with mRNA encoding the desired antigen.^4,28,30^ The DCs then present the antigen to the immune system, leading to a robust immune response. This approach has shown promise in preclinical studies for the treatment of cancers and infectious diseases. In recent years, lipid-based nanoparticles (LNPs) and polyplexes/polymeric nanoparticles have been two of the most commonly used mRNA vaccine delivery systems.^38,41^

LNPs are extensively utilized as delivery systems for mRNA vaccines owing to their biocompatibility, stability, and ability to protect mRNA from degradation.^4,28,38,41,53,54^ LNPs can be categorized based on the nature of their lipid components, surface charge, and surface modifications.^41,55^ One category is cationic LNPs, with positively charged lipid components interacting with the negatively charged phosphate backbone of mRNA, facilitating the latter’s delivery into target cells.^38^ Previous studies have provided evidence of the efficacy of ionizable LNP-based vaccines against different infectious diseases.^4,55^ Ionizable LNPs hold great potential as a delivery vehicle for mRNA-based vaccines.^4,38,41^ These nanoparticles are composed of a central core of mRNA enclosed by a lipid bilayer that incorporates ionizable lipids, which allow effective mRNA encapsulation and protection from degradation in the extracellular milieu. Moreover, the ionizable lipids are instrumental in promoting the endosomal release and cytoplasmic transport of the mRNA cargo, which is pivotal for efficient protein expression. Polyethylene glycol (PEG)-ylated LNPs have a hydrophilic coating of PEG on their surface, which enhances biocompatibility and reduces toxicity.^28,38^

Polyplexes and polymeric nanoparticles are versatile delivery systems that have been extensively studied for mRNA vaccines. Polyplexes are formed by electrostatic interactions between positively charged polymers, such as polyethyleneimine, and negatively charged mRNA molecules.^14^ These effectively protect mRNA from degradation, facilitate cellular uptake and enhance immunogenicity due to their cationic charge.^14^ Polymeric nanoparticles can be formed from various polymers, including poly lactic-co-glycolic acid and PEG, and mRNA can be encapsulated through multiple mechanisms, involving electrostatic interactions, hydrophobic interactions, and covalent bonding.^41,56^ These have lower immunogenicity and toxicity than polyplexes and LNPs and can be engineered to enhance their stability and targeting specificity.^56,57^ However, their transfection efficiency may be lower than that of LNPs, and their production can be more complex and costly.

In general, the choice of delivery system depends on several factors, including the specific characteristics of the mRNA vaccine and the desired transfection efficiency, safety, stability, and target specificity.

### mRNA vaccine adjuvants

Immunogenicity modulation is a nonnegligible issue in mRNA vaccine development. Although in vitro transcriptional mRNA has shown some self-adjuvant potential, it is typically not enough to elicit comprehensive protective immunity and requires intensified repeated/booster regimens for optimal effectiveness.^58^ Multiple strategies have been applied for adjuvants of mRNA vaccines to regulate their immunogenicity. TriMix is a combination of mRNAs that encode three distinct immune-stimulating proteins: CD40 ligand (CD40L), CD70, as well as constitutively active Toll-like receptor 4 (TLR4).^59–61^ Due to its ability to improve DC activation and enhance the elicitation of CD8^+^ T-cell responses, TriMix has been incorporated into numerous vaccination studies. Moreover, the utilization of cationic lipids is widely recognized for its ability to improve RNA uptake and facilitate its endosomal escape, resulting in increased adjuvant activity for mRNA vaccines.^62,63^ Furthermore, the incorporation of a synthetic mRNA sequence with a polymeric carrier has been shown to enhance the adjuvanticity of various subunit vaccines.^64^ CureVac has developed RNActive® vaccines, which demonstrate inherent self-adjuvant activity by incorporating naturally occurring nucleotides complexed with protamine.^65,66^ The co-delivery of this mRNA construct has been proven to significantly amplify B and T-cell responses along with the amplification of subpopulations (e.g., Th1 and Th2 cells) and pre-germinal center B cells. However, the adjuvant properties of these strategies usually activate type I interferon (IFN-I), which might cause the suppression of protein translation as well as CD8^+^ T-cell activation.^67,68^ To overcome this limitation, a hybrid nanoparticle system comprising a poly lactic-co-glycolic acid core and a lipid shell has been developed for simultaneous delivery of mRNA and a hydrophobic TLR7 adjuvant (gardiquimod). Poly lactic-co-glycolic acid facilitates the integration of the adjuvant within the nucleus, whereas the lipid shell enables the loading of mRNA via electrostatic interactions. This approach has demonstrated potent immune responses targeting specific antigens and highly effective antitumour activities.^69^

## mRNA vaccines in infectious diseases

**Fig. 3:**
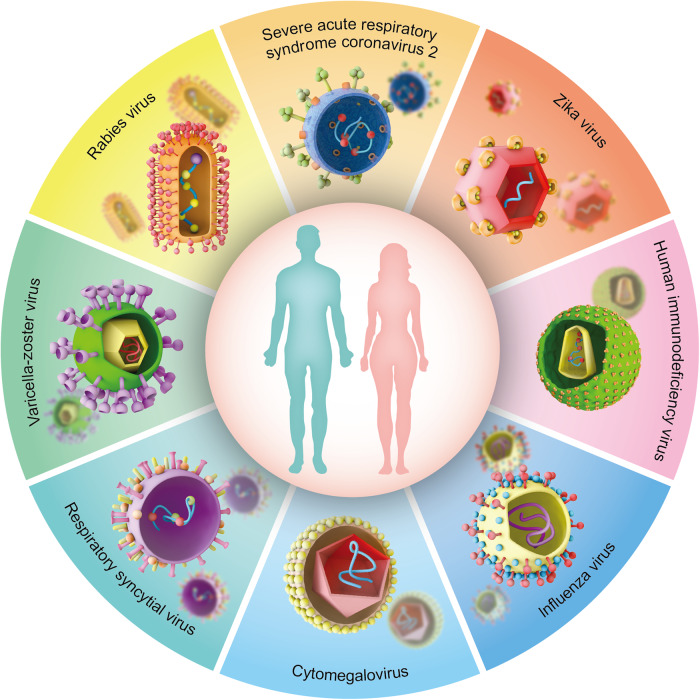
Landscape of mRNA vaccines in infectious diseases. mRNA vaccines have been developed against multiple infectious diseases to date, including severe acute respiratory syndrome coronavirus 2, zika virus, human immunodeficiency virus, influenza virus, cytomegalovirus, respiratory syncytial virus, varicella-zoster virus, and rabies virus. This figure is created using Adobe Illustrator and integrates the current literature-based knowledge

mRNA vaccines are applied as prophylaxis against infectious diseases by encoding disease-specific antigens. To date, many preclinical and clinical trials using mRNA vaccines to induce antiviral immunity have been performed in multiple infectious diseases, including severe acute respiratory syndrome coronavirus 2, zika virus, human immunodeficiency virus, influenza virus, cytomegalovirus, respiratory syncytial virus, varicella-zoster virus, and rabies virus (Table 1 and Fig. 3).

**Table 1:** Clinical trials for mRNA vaccines in infectious diseases

Catalogue	NCT number	mRNA vaccine	Encoded antigen	Vehicle	Phase	Status
Severe acute respiratory syndrome coronavirus 2	NCT04283461	mRNA-1273	Full-length, prefusion stabilized spike protein	Lipid Nanoparticle	Phase I	Completed
	NCT04470427	mRNA-1273	Full-length, prefusion stabilized spike protein	Lipid Nanoparticle	Phase III	Completed
	NCT04368728	BNT162b1, BNT162b2	Spike glycoprotein receptor-binding domain	Lipid Nanoparticle	Phase II/III	Completed
	NCT04480957	LUNAR-COV19	Spike protein with two proline substitutions	Lipid Nanoparticle	Phase I/II	Completed
	NCT04860258	CVnCoV	Spike protein with two proline substitutions	Lipid Nanoparticle	Phase III	Terminated
	NCT04847102	ARCoV	Spike glycoprotein receptor-binding domain	Lipid Nanoparticle	Phase III	Recruiting
	NCT05364047	LVRNA009	Spike glycoprotein receptor-binding domain	Lipid Nanoparticle	Phase I	Recruiting
Zika virus	NCT03014089	mRNA-1325	Glycoproteins of Zika virus	Unknown	Phase I	Completed
	NCT04917861	mRNA-1893	Glycoproteins of Zika virus	Lipid Nanoparticle	Phase II	Active, not recruiting
Human immunodeficiency virus	NCT02042248	AGS-004	Human immunodeficiency virus-1 antigen	DC	Phase I	Completed
	NCT00381212	AGS-004	Human immunodeficiency virus-1 antigen	DC	Phase II	Completed
Influenza	NCT03076385	VAL-506440	Membrane-bound form of the hemagglutinin glycoprotein	Lipid Nanoparticle	Phase I	Completed
	NCT03345043	VAL-339851	Membrane-bound form of the hemagglutinin glycoprotein	Lipid Nanoparticle	Phase I	Completed
	NCT05333289	mRNA-1030, mRNA-1020, mRNA-1010	Unknown	Unknown	Phase I/II	Recruiting
Cytomegalovirus	NCT03382405	mRNA-1647, mRNA-1443	Unknown	Lipid Nanoparticle	Phase I	Completed
Respiratory syncytial virus	NCT05127434	mRNA-1345	Unknown	Unknown	Phase II/III	Recruiting
Rabies virus	NCT03713086	CV7202	Glycoprotein of Rabies virus	Lipid Nanoparticle	Phase I	Completed
	NCT02241135	CV7202	Glycoprotein of Rabies virus	Lipid Nanoparticle	Phase I	Completed

### mRNA vaccines against severe acute respiratory syndrome coronavirus 2

Since the beginning of 2021, severe acute respiratory syndrome coronavirus 2 (SARS-CoV-2) has infected countless people as well as caused millions of deaths worldwide. The majority of SARS-CoV-2 infections do not pose a life-threatening risk to individuals without preexisting diseases; however, in cases of severe infection, uncontrolled immune responses can be triggered in the lungs, destroying epithelial cells and alveoli, causing pulmonary edema, a dangerous increase in vascular permeability and death.^70,71^ The spike protein, which is found on the surface of SARS-CoV-2, facilitates the virus’s entry into host cells by binding to the angiotensin-converting enzyme 2 receptors on the surface of the host cells.^72^ Therefore, the spike protein represents a prime target for SARS-CoV-2 mRNA vaccines encoding either the receptor-binding domain or the full-length spike protein. To date, two mRNA vaccines designed to target the spike protein of the coronavirus disease 2019 (COVID-19) have gained approval and widespread usage globally. These vaccines include mRNA-1273 developed by Moderna and BNT162b2 developed by BioNTech/Pfizer. Meanwhile, several other mRNA vaccines targeting the spike protein are currently undergoing clinical trials assessing their safety and efficacy.

The initial phase I clinical study for COVID-19 vaccine was conducted on mRNA-1273, which was developed by Moderna. In the formulation of LNPs to encapsulate modified mRNA, the ionizable lipid SM-102 was utilized. The mRNA sequence was modified with N1-methylpseudouridine encoding the spike protein of SARS-CoV-2 with two proline substitutions (S-2P), which induce the prefusion conformation. A study performed by Corbett et al. in 2020 exhibited the administration of mRNA-1273 triggered potent humoral and cellular immunity against original and mutant (D614G) SARS-CoV-2 in preclinical models.^73,74^ The administration of the vaccine effectively provided protection to mice, preventing SARS-CoV-2 infection in the nasal passages and lungs without evident adverse effects or pathological changes in the respiratory system. The following phase I clinical trial conducted in July 2020 validated the safety and efficacy of mRNA-1273 in humans. The geometric mean titers of anti-S-2P neutralizing antibodies after the second vaccination were 299,751, 782,719, and 1,192,154 in patients who received 25 μg, 100 μg, or 250 μg of mRNA-1273, respectively, suggesting a robust humoral immune response in participants. A robust T cell-mediated cytokine response was also detected.^75^ The majority of the reported adverse events following vaccination were mild to moderate in nature. These included symptoms such as headache, chills, injection site pain, fatigue, and myalgia, with more than half of the participants experiencing these effects. Patients who received the 250 μg dose exhibited a higher incidence (21%) of severe adverse events, particularly when the second vaccine was administered.^75^ In September 2020, elderly participants were also involved in the trial, without any trial-limiting adverse effects observed.^76^ The phase III randomized, placebo-controlled study was carried out at multiple medical centers in the United States from July to December 2020 and involved 30,420 volunteers, and the results showed that SARS-CoV-2 infection was diagnosed in 185 participants in the control group, while this infection was diagnosed in only 11 patients in the vaccinated group. mRNA-1273 demonstrated a 94.1% effectiveness against SARS-CoV-2 infection and a 100% efficacy against severe COVID-19 disease, with a transient and mild local and systemic reaction induced by mRNA-1273.^77^ In 2022, Creech et al. evaluated mRNA-1273 in 6 to 11-year-old children in phase II/III trial.^78^ In the first phase of the trial, 751 children were administrated 50 μg or 100 μg doses of the mRNA-1273 vaccine. On the basis of the results of safety and immunogenicity, the 50 μg dose level was chosen for the second phase of the trial. The second phase of the trial involved the random administration of two injections of mRNA-1273 (50 μg each) or placebo to a group of 4,016 children, and these participants were then monitored for a median duration of 82 days after the first injection. At this dose level, the observed adverse events were primarily mild and temporary, with injection-site pain, headache, and fatigue being the most commonly reported. As of the data-cut-off date, no severe side effects associated with the vaccine were reported, such as multisystem inflammatory syndrome, myocarditis, or pericarditis. At 1 month following the second injection, children receiving mRNA-1273 at a 50 μg level exhibited a neutralizing antibody titer of 1610, whereas young adults receiving the 100 μg level had a titer of 1300. Serologic responses were observed in a minimum 99.0% of participants within both age cohorts. At a point when the dominant circulating variant was Delta, the evaluated vaccine effectiveness against COVID-19 occurring 14 days or more after the initial injection was 88.0%. Overall, mRNA-1273 shows promising anti-COVID-19 efficacy, significantly protecting individuals from COVID-19.

BNT162b1 and BNT162b2 are two COVID-19 mRNA vaccines developed by BioNTech and Pfizer. These vaccines are enclosed within LNPs and formulated utilizing Acuitas Therapeutics’ ionizable lipid ALC-0315. The mRNA in these vaccines is nucleoside-modified, with all uridines substituted by N1-methylpseudouridine, which enhances mRNA translation. BNT162b1 encoded a secreted S glycoprotein receptor-binding domain (RBD) protein, while BNT162b2 encoded the S-2P protein. The relevant phase I clinical study was performed in April 2020, and healthy participants in distinct groups were treated with either placebo or two doses of one of the two vaccines mentioned above at differential doses (10 μg, 20 μg, 30 μg, and 100 μg) with a 21-day interval. Both BNT162b1 and BNT162b2 resulted in a strong serologic response against the virus in a dose-dependent manner, especially following the second dose. The highest level of neutralizing antibodies was detected on day 35, which was 14 days after the second dose.^79,80^ Although both BNT162b1 and BNT162b2 elicited a potent and robust immune response, BNT162b2 was related to a lower risk of systematic adverse effects than BNT162b1, especially in elderly participants, leading to the selection of BNT162b2 to be used in a broader cohort enrolled in phase III clinical study involving 43,448 participants enrolled from April to December 2020.^80^ A total of 21,720 participants received BNT162b2, while 21,728 participants received a placebo. The results revealed that eight patients in the vaccinated group were diagnosed with SARS-CoV-2 infection, whereas 162 patients in the placebo group were found to be infected, suggesting that the efficacy of BNT162b2 was 95%. Among the infected patients, 10 were severely ill, with nine of them belonging to the placebo group and one to the vaccinated group.^81^ In addition, BNT162b2 vaccination elicited a strong and enduring response of T follicular helper cells in humans.^82^ In a study performed by Muik et al. in 2022, sera from 51 individuals receiving two or three doses of BNT162b2 vaccine were tested against original type, Beta, Delta, or Omicron pseudoviruses.^83^ After two doses, the neutralizing titers against the Omicron variant showed a reduction of more than 22-fold compared to the titers against the wild-type. One month after receiving the third vaccine dose, the neutralizing titers against the Omicron variant enhanced by 23-fold compared to the titers after two doses, which were analogous to the levels of neutralizing titers against the original type observed after two doses. Together, BNT162b2 is associated with superior safety and exhibits potent efficiency against COVID-19, which is also effective in the context of variants.

Multiple trials have compared the efficacy of mRNA-1273 and BNT162b2 vaccines. In a study performed by Wang et al. in 2022, a comparison was made between mRNA-1273 and BNT162b2 vaccines in terms of breakthrough SARS-CoV-2 infections, hospitalizations, and deaths during the period when the delta variant was predominant.^84^ The monthly incidence rate of breakthrough infections showed a gradual increase from July to November 2021 in both the BNT162b2 cohort and the mRNA-1273 cohort. However, the incidence rate was elevated in the BNT162b2 cohort compared with the mRNA-1273 cohort. Specifically, in November, the incidence rate reached 2.8 cases per 1000 person-days in the BNT162b2 cohort as well as 1.6 cases per 1000 person-days in the mRNA-1273 cohort. After conducting matching analysis, it was found that the mRNA-1273 cohort, consisting of 62,584 individuals, exhibited a markedly decreased hazard for breakthrough infections relative to BNT162b2 cohort, which also included 62,584 individuals. Among the patients who experienced breakthrough infections, it was observed that individuals receiving the mRNA-1273 vaccine were generally older compared to those receiving the BNT162b2 vaccine. There were also differences in terms of sex, racial and ethnic composition, and the presence of comorbidities and adverse social determinants of health. After conducting the matching analysis, these differences were no longer found to be statistically significant. Among the individuals receiving the mRNA-1273 vaccine, the 60-day hospitalization risk was 12.7%, with 392 out of 3,078 recipients requiring hospitalization. In comparison, among those receiving the BNT162b2 vaccine, the 60-day hospitalization risk was slightly higher at 13.3%, with 2,489 out of 18,737 recipients requiring hospitalization. In terms of mortality, the 60-day mortality rate for mRNA-1273 recipients was 1.14%, with 35 out of 3,078 individuals experiencing mortality. For BNT162b2 recipients, the 60-day mortality rate was 1.10%, with 207 out of 18,737 individuals experiencing mortality. Among the matched cohorts consisting of 3,054 individuals in each group, recipients of the mRNA-1273 vaccine showed a decreased risk of 60-day hospitalizations compared to those of the BNT162b2 vaccine. Similarly, a study performed by Dickerman et al. examined the efficacy of the BNT162b2 and mRNA-1273 vaccines in a group of U.S. Veterans.^85^ Each vaccine group consisted of 219,842 individuals. During the 24-week follow-up period, which was characterized by the predominance of the alpha variant, the assessed risk of documented infection was 5.75 events per 1000 individuals in the BNT162b2 group and 4.52 events per 1000 individuals in the mRNA-1273 group. The additional quantity of events per 1000 individuals for BNT162b2 relative to mRNA-1273 was 1.2 for documented infection, 0.44 for symptomatic COVID-19, 0.55 for hospitalization for COVID-19, 0.10 for ICU admission for COVID-19, and 0.02 for mortality from COVID-19. The relative excess risk of documented infection for BNT162b2 compared to mRNA-1273 over a 12-week follow-up period, during which the delta variant was predominant, was 6.54 events per 1000 persons. Together, relative to people vaccinated with mRNA-1273, those with BNT162b2 show lower rates of symptoms, hospitalization, ICU admission, and death, despite the higher infection rate.

Multiple studies have been conducted to explore the utilization of mRNA-1273 or BNT162b2 in the context of SARS-CoV-2 variants. Both mRNA-1273 and BNT162b2 vaccines demonstrated enhanced potency and breadth in memory B-cell response, effectively triggering neutralizing immunity against the SARS-CoV-2 omicron variant.^86–89^ Fabiani et al. conducted a study to assess the effectiveness of mRNA vaccines and the waning protection against SARS-CoV-2 infection as well as severe COVID-19 in a population of 33,250,344 individuals aged 16 years and above receiving their initial dose of either BNT162b2 or mRNA-1273 vaccine and showed no better diagnosis of SARS-CoV-2 infection in Italy.^90^ In the period characterized by the prevalence of the delta variant, vaccine efficacy against SARS-CoV-2 infection notably declined from 82% at 3–4 weeks to 33% at 27–30 weeks after the second dose. In the same time intervals, the effectiveness of the vaccines against severe COVID-19 also experienced a decline, although the decline was not as pronounced, from 96% to 80%. At 27–30 weeks after the second dose of the vaccine, high-risk individuals, including those aged 80 years and older, as well as those aged 60–79 years, did not appear to be adequately protected against infection. Abu-Raddad et al. investigated the impact of mRNA vaccine boosters on SARS-CoV-2 omicron infection in 2,239,193 individuals administrated with a minimum of two doses of the BNT162b2 or mRNA-1273 vaccine in Qatar.^91,92^ After 35 days of follow-up, the cumulative incidence of symptomatic omicron infection among individuals who received the BNT162b2 vaccine was 2.4% in the booster cohort and 4.5% in the nonbooster cohort. The effectiveness of the booster dose in protecting against symptomatic omicron infection, when compared to the initial primary series, was determined to be 49.4%. The efficacy of the booster dose in reducing COVID-19-related hospitalization and mortality owing to omicron infection, compared to the initial vaccine series, was estimated to be 76.5%. The effectiveness of the BNT162b2 booster dose in decreasing symptomatic infection with the delta variant, relative to the initial vaccine series, was estimated to be 86.1%. Among individuals who received the mRNA-1273 vaccine, the cumulative incidence of symptomatic omicron infection was 1.0% in the booster cohort and 1.9% in the nonbooster cohort after 35 days. The effectiveness of the mRNA-1273 booster dose in reducing symptomatic omicron infection, relative to the primary vaccine series, was estimated to be 47.3%. In addition, Accorsi et al. investigated the relation between 3 doses of BNT162b2 or mRNA-1273 and symptomatic infection resulted from the SARS-CoV-2 omicron and delta variants.^93^ Among the reported cases, 18.6% (*n* = 2,441) of omicron cases, 6.6% (*n* = 679) of delta cases, and 39.7% (*n* = 18,587) of controls had received three doses of mRNA vaccines. Furthermore, 55.3% (*n* = 7245) of cases, 44.4% (*n* = 4570) of delta cases, and 41.6% (*n* = 19,456) of controls had received two doses of mRNA vaccines. Lastly, 26.0% (*n* = 3412) of cases, 49.0% (*n* = 5044) of delta cases, and 18.6% (*n* = 8721) of controls were reported to be unvaccinated. After adjusting for relevant factors, the odds ratio for receiving three doses compared to being unvaccinated was 0.33 for omicron cases and 0.065 for delta cases. Similarly, the odds ratio for three vaccine doses compared to two doses was 0.34 for omicron cases and 0.16 for delta cases. Grewal et al. conducted a study to estimate the marginal efficacy of a fourth dose relative to a third dose, as well as the overall vaccine effectiveness of BNT162b2 and mRNA-1273 against any infection, symptomatic infection, and severe outcomes (hospital admission or death) associated with the omicron variant of SARS-CoV-2.^94^ When comparing a fourth dose of the vaccine (with 95% of recipients receiving mRNA-1273) administered seven days or more after vaccination to a third dose received 84 or more days prior, the marginal effectiveness was estimated to be 19% against any infection, 31% against symptomatic infection, and 40% against severe outcomes (hospital admission or death). The effectiveness of the vaccine in individuals receiving the vaccine, as compared with those who were unvaccinated, showed a progressive increase with each additional dose. Specifically, for a fourth dose, the effectiveness was observed to be 49% against overall infection, 69% against symptomatic infection, and 86% against severe outcomes. Lauring et al. assessed clinical severity and efficacy of BNT162b2 and mRNA-1273 vaccines against COVID-19 caused by the omicron, delta, and alpha variants of the SARS-CoV-2 virus.^95^ The study involved a total of 5,728 individuals with COVID-19 and 5,962 individuals without COVID-19 in the United States. Among individuals who received two vaccine doses, the rates were 85% for the alpha variant, 85% for the delta variant and 65% for the omicron variant. For individuals who received three vaccine doses, the rate was 94% against the delta variant and 86% against the omicron variant. In-hospital mortality was 7.6% (81/1060) for alpha, 12.2% (461/3788) for delta, and 7.1% (40/565) for omicron. For unvaccinated patients with COVID-19 who were hospitalized, the severity of illness, as measured by the WHO clinical progression scale, was found to be elevated for the delta variant compared to the alpha variant, with an adjusted proportional odds ratio of 1.28. Conversely, the severity of illness was lower for the omicron variant relative to the delta variant, with an adjusted proportional odds ratio of 0.61. Compared with unvaccinated patients, vaccinated patients exhibited lower severity of illness for each variant, including the alpha variant (adjusted proportional odds ratio 0.33), the delta variant (0.44), and the omicron variant (0.61). Together, mRNA-1273 and BNT162b2 also show protective efficacy in the context of COVID-19, with BNT162b2 showing superior efficacy against COVID-19 variants. Although this effect declines over time, further booster doses can partially reverse this phenomenon, representing a strategy against COVID-19 variants.

On August 31, 2022, the U.S. Food and Drug Administration (FDA) has updated the emergency use authorizations for the Moderna COVID-19 Vaccine and the Pfizer-BioNTech COVID-19 Vaccine to allow for the use of bivalent formulations as a single booster dose (derived from https://www.fda.gov/news-events/press-announcements/coronavirus-covid-19-update-fda-authorizes-moderna-pfizer-biontech-bivalent-covid-19-vaccines-use). The updated boosters include two mRNA elements derived from the SARS-CoV-2 virus. These bivalent formulations consist of one component from the initial type of the virus and another element owned by the BA.4 and BA.5 lineages of the omicron variant. The recommended interval for administering the booster dose is at least 2 months after the primary or previous booster vaccination. The Moderna COVID-19 Vaccine, Bivalent, has been approved as a standalone booster shot for individuals who are 18 years old or older. The Pfizer-BioNTech COVID-19 Vaccine, Bivalent, has been authorized as a single booster dose for people who are 12 years old and above. The FDA conducted an evaluation of immune response data involving around 600 adults aged 18 and above who had already received two doses of the primary series and an additional booster dose of the monovalent Moderna COVID-19 vaccine. These individuals were administered a second booster dose of the monovalent Moderna COVID-19 vaccine or Moderna’s experimental bivalent COVID-19 vaccine, which includes the original strain and the BA.1 Omicron variant, minimum 3 months after their initial booster shot. After a period of 28 days, the group receiving the bivalent vaccine demonstrated a superior immune response against the BA.1 Omicron variant compared to the group receiving the monovalent Moderna COVID-19 vaccine. Since the bivalent (original and omicron BA.1) and monovalent Moderna COVID-19 vaccines are manufactured using the same process, the safety data obtained from the bivalent vaccine are relevant and applicable to the monovalent Moderna COVID-19 vaccine. To assess the efficacy of a single booster shot of the Pfizer-BioNTech COVID-19 vaccine, Bivalent, for individuals aged 12 and above, the FDA examined immune response data from around 600 individuals over the age of 55 previously receiving a two-dose primary series and an additional booster dose using the monovalent Pfizer-BioNTech COVID-19 vaccine. These individuals were administered a second booster dose of the monovalent Pfizer-BioNTech COVID-19 vaccine or Pfizer-BioNTech’s experimental bivalent COVID-19 vaccine, which includes the original strain and the BA.1 Omicron variant, between 4.7 and 13.1 months after their initial booster dose.

After 1 month, the immune responses against BA.1 Omicron variant in the group receiving the bivalent vaccine were found to be superior to the immune responses observed in the group receiving the monovalent Pfizer-BioNTech COVID-19 vaccine. Because the bivalent vaccine and the monovalent vaccine are manufactured using the same process, the safety data are relevant to the Pfizer-BioNTech COVID-19 vaccine. Following this approval, the FDA has revised the emergency use authorizations for the Moderna COVID-19 vaccine and the Pfizer-BioNTech COVID-19 Vaccine, eliminating the usage of the monovalent Moderna and Pfizer-BioNTech COVID-19 vaccines for booster doses in people 18 years and older and 12 years and older, respectively. These monovalent vaccines are still authorized for application as a primary series for individuals aged 6 months and above, as outlined in their respective letters of authorization. The Pfizer-BioNTech COVID-19 vaccine is presently authorized for a single booster shot for people who are 5 to 11 years old, minimum 5 months after finishing a primary series of the Pfizer-BioNTech COVID-19 vaccine. Overall, the bivalent vaccine represents a new step in mRNA vaccines against COVID-19.

One of the problems with mRNA vaccines is the requirement of extremely low-temperature storage, which limits their application in areas with poor conditions and low economic levels. CVnCoV is a chemically unmodified mRNA vaccine encoding S-2P developed by CureVac AG, which is stable at +5 °C for at least 3 months and was first reported in April 2020. Preclinical models using CVnCoV revealed that this vaccine induced robust humoral responses as well as strong T-cell responses with potent induction of IFN-γ^+^ TNF^+^ T cells. In addition, the animals infected with SARS-CoV-2 with the spike D614G substitution 4 weeks after vaccination showed no detectable virus in the lower respiratory tract after a dose of 10 μg. Moreover, CVnCoV decreased the histopathological alterations in the lungs of mice infected with SARS-CoV-2.^96^ The phase I clinical trial performed in June 2020 exhibited that two doses of CVnCoV administered to individuals were safe and well tolerated. CVnCoV significantly increased the levels of IgG antibodies to S-protein, as well as RBD in a dose-dependent manner, and the median antibody titers after two 12 μg doses of CVnCoV were similar to those in the serum from patients with COVID-19.^97^ Therefore, a dose of 12 μg was chosen for the phase II/III trial. The randomized phase IIb/III clinical trial was conducted in 47 centers all over the world from December 2020 to April 2021. After more than 40 days of observation, 83 patients among the 12,851 in the CVnCoV group were diagnosed with SAR-CoV-2 infection, and 145 patients among the 12,211 in the placebo group were diagnosed with SAR-CoV-2 infection; the overall vaccine efficacy of only 48.2% was partly owing to the presence of SARS-CoV-2 variants.^97^ The same year, CureVac AG announced its second-generation mRNA vaccine CV2CoV, which possesses optimized noncoding regions to improve the level of the targeted antigen. CV2CoV induced higher titers of neutralizing antibodies and stronger T-cell responses in nonhuman primates than CVnCoV. Moreover, the findings of the challenge assay displayed that CV2CoV induced stronger protection with lower viral loads in both the upper and lower respiratory tract. Clinical trials have been planned and will be performed soon in the future.^98^

ARCoV is an LNP mRNA vaccine encoding an RBD protein that was developed by Abogen in 2020. ARCoV mRNA-LNP used in preclinical mouse models triggered high titers of neutralizing antibodies and strong T-cell responses against SARS-CoV-2, with significantly increased IFN-γ and TNF-α secreted by virus-specific CD4^+^ and CD8^+^ T cells. Further infection with SARS-CoV-2 in vaccinated mice showed that ARCoV protected mice from SARS-CoV-2 infection with no measurable viral RNA in the lungs of the vaccinated mice.^99^ Two doses of ARCoV in nonhuman primate models triggered potent humoral responses characterized by elevated titers of neutralizing antibodies and strong cellular responses against SARS-CoV-2 in cynomolgus macaques. The challenge assay revealed no detectable viral small guide RNAs in the trachea and lung lobes of all the vaccinated cynomolgus macaques, while robust viral replication was present in macaques receiving a placebo treatment. These results suggested the ability of ARCoV to prevent SARS-CoV-2 replication in the lower respiratory tract.^100^ A phase III clinical study was initiated in April 2021 in multiple centers in Indonesia and Mexico (NCT04847102). Further exposure of clinical results is required to assess the effectiveness of this mRNA vaccine.

LUNAR-COV19 is a self-replicating mRNA vaccine encoding an S-2P antigen developed by Arcturus in 2020, with the aim of offering robust immunity with a single low-dose administration.^101^ LUNAR-COV19 used in preclinical models induced a robust T-cell response with an expanded CD44^+^CD62L^-^ effector/memory subset, enhanced the proportion of IFN-γ^+^ CD8^+^/CD4^+^ T cells, as well as resulted in potent humoral responses with high titers of neutralizing antibodies. Eighty percent of mice treated with 10 mg LUNAR-COV19 exhibited PRNT50 titers >320 at 30 days after vaccination. The human ACE2 transgenic C57BL/6 mouse model was used for the challenge assay, revealing unchanged weight and no clinical sign in the vaccinated mice after infection with original type SARS-CoV-2, while mice that received placebo showed an increased clinical score and a significant decrease in weight after infection.^101^ The assessment of the viral load revealed no detectable SARS-CoV-2 RNA in both lungs of the vaccinated mice compared to the mice treated with a placebo. LUNAR-COV19 used in phase II clinical study (NCT04480957) was well tolerated, and increased neutralizing antibody levels were observed in the enrolled patients. Further investigation is required for the broader application of this vaccine.

Together, the approvals of mRNA vaccines not only protect numerous individuals from COVID-19 but also provide valuable experience for the development of mRNA vaccines against other diseases. Of note, although various anti-SARS-CoV-2 mRNA vaccines have been prepared and used in humans, there are still problems that have not been solved, and the mechanism of action is still unclear. For example, the duration of the protection provided by the mRNA vaccine in humans against COVID-19, as well as how to increase the levels of IgA antibodies, which are those that mainly protect the upper respiratory tract, are not yet known. How to reduce the rate of adverse effects, as the incidence of systemic adverse events induced by mRNA vaccines is still higher compared to those triggered by inactivated virus vaccines or protein subunits, as demonstrated in previous clinical trials. Long-term monitoring might provide more detailed and useful information leading to the safe and extensive application of mRNA vaccines.

### mRNA vaccines against Zika virus

Zika virus (ZIKV) is an RNA virus with a positive sense, single-stranded genome measuring 11 kilobases in length.^102^ People infected with ZIKV often develop fever, headache, rash, malaise, and conjunctivitis that last between two and seven days. However, its tropism for progenitor neural cells causes neurodevelopmental birth defects and congenital malformation in a limited number of instances.^103^ Preventive vaccination is the only option against the complications of ZIKV infection, as no drug against this virus is available.^104^ Membrane and envelope proteins are common antigens for mRNA vaccines against ZIKV. To date, several ZIKV vaccines developed on the basis of the mRNA platform have been tested in preclinical models. In 2017, Pardi et al. designed an LNP-enclosed mRNA vaccine encoding the glycoproteins of the membrane and envelope of ZIKV.^105^ The administration of 30 μg mRNA vaccine in C57BL/6 mice elicited a robust immune response without any inflammation or other adverse events. The ZIKV reporter viral particle assay showed that the mean neutralizing IgG against the ZIKV virus peaked at 8 weeks after vaccination and was stable until 12 weeks after administration. Strong E-protein-specific CD4^+^ T-cell responses were also observed as evidenced by robust intracellular production of IL-2, TNF-α, and IFN-γ. Moreover, a challenge study showed that mice and nonhuman primates treated with the mRNA vaccine exhibited protection against ZIKV infection.^105^ The same year, Richner et al. developed an LNP-enclosed mRNA vaccine encoding both original type and variant ZIKV membrane glycoproteins. Two doses of the mRNA vaccine potentiated the serum-neutralizing responses against ZIKV and protected mice against ZIKV infection. The efficacy of the mRNA vaccine was also assessed in a mouse pregnancy model. The vaccinated mice were infected with ZIKV at embryo day six, and the results exhibited two doses of mRNA vaccine significantly reduced the levels of viral RNA in both fetal and placental tissues.^106,107^ Although the results of the mRNA ZIKV vaccine in preclinical studies are promising, further human clinical trials are needed. However, clinical trials for these vaccines in pregnant women are undermined by ethical issues.

### mRNA vaccines against human immunodeficiency virus

Human immunodeficiency virus (HIV) is a member of the Lentivirus genus of the retroviridae family and is divided into two types: HIV-1 and HIV-2.^108,109^ HIV causes acquired immune deficiency syndrome (AIDS), which infects 75 million people worldwide, causing more than 32 million AIDS-related deaths (derived from Global HIV and AIDS statistics, 2019). No effective preventive vaccine exists despite 30 years of research, primarily because of the significant antigenic diversity of the protein found in the HIV envelope and its dense "glycan shield that hides the epitope of the crucial envelope protein. Multiple mRNA vaccines have been investigated in clinical studies to date. In 2016, Gandhi et al. used mRNA-transfected autologous DCs to stimulate the immune response against HIV-1.^110^ Fifteen patients were involved in the trial and randomly assigned to two separate groups that received DC mRNA vaccines encoding HIV-1 antigen or placebo. The proliferative response of CD4^+^ T cells to HIV-1 Gag was significantly enhanced by DC mRNA vaccines, with a 3.4-fold increase compared to that in participants administrated with a placebo. However, no significant release of IFN-γ was detected, and the increase in the CD8^+^ T cell proliferative response was transient.^110^ In 2017, Jong et al. developed an HIV mRNA immunogen based on conserved targets of effective antiviral T-cell responses against HIV.^111^ The phase I trial using increasing doses of this vaccine showed that it was safe and well tolerated.^111^ Despite these encouraging findings, the phase II clinical study in the same year was stopped due to the production of insufficient immunogenicity by the vaccine. In 2020, Gay et al. combined AGS-004, a DC mRNA vaccine, with the latency-reversing agent vorinostat and evaluated the effect on the HIV reservoir. The aim of this combination therapy was to disrupt the virological latency by vorinostat and to deplete cells expressing HIV antigens and clear the HIV reservoir by the mRNA vaccine. However, although the combination of AGS-004 and vorinostat was safe and well tolerated, no substantial impact on the immune response against HIV was observed, and the frequency of resting CD4^+^ T-cell infection was stable throughout the entire treatment in all participants.^112^ A mRNA vaccine concurrently expressed membrane-anchored HIV-1 envelope (Env) and simian immunodeficiency virus (SIV) Gag proteins, was created to generate of virus-like particles.^113^ This vaccine formulation elicited the production of antibodies with broad neutralizing capabilities against HIV-1 and demonstrated a reduction in the risk of infection in rhesus macaques. Rhesus macaques were initially primed with an mRNA vaccine containing a transmitted founder clade-B env protein lacking the N276 glycan. Multiple booster immunizations were administered to the rhesus macaques using autologous Envs that were repaired with the missing glycan and subsequently with bivalent heterologous Envs from clades A and C. The vaccination regimen described was highly effective in inducing a strong immune response, resulting in the production of neutralizing antibodies against the most prevalent (tier-2) strains of HIV-1 and robust anti-Env CD4^+^ T cell responses. Upon conducting multiple low-dose mucosal challenges with heterologous tier-2 simian-HIV AD8, the vaccinated animals demonstrated a 79% per-exposure risk decrease. The findings suggest that the multiclade Env-Gag virus-like particle mRNA platform holds promise as a potential method for developing an HIV-1 vaccine. Of note, a biotech firm, in collaboration with the nonprofit partner IAVI (International AIDS Vaccine Initiative), has initiated a phase I clinical trial for an investigational mRNA HIV vaccine (https://investors.modernatx.com/news/news-details/2022/IAVI-and-Moderna-Launch-Trial-of-HIV-Vaccine-Antigens-Delivered-Through-mRNA-Technology/default.aspx). The vaccine candidate in question utilizes a prime and boost strategy to elicit targeted B-cell responses with the objective of generating broadly neutralizing antibodies against HIV. The antigens employed in the vaccine were developed as proteins by scientists at IAVI. They previously investigated the prime antigen in an adjuvanted protein-based vaccine, inducing the desired B-cell response in 97% of trial participants. Notably, the development of the mRNA HIV vaccine is still in its initial stage. More research is needed to optimize this treatment strategy for long-lasting immune responses. The studies focus on the simultaneous administration of drugs that help reactivate the HIV reservoir to make it visible to the immune system and may eventually improve the efficacy of the mRNA HIV vaccine.

### mRNA vaccines against influenza virus

Influenza viruses are members of the Orthomyxoviridae family composed mainly of four types of influenza viruses: types A, B, C, and D; among them, types A and B are clinically associated with human diseases.^114,115^ The typical target of the mRNA vaccine against influenza virus is the glycoprotein haemagglutinin (HA) on the surface of the virus since it mediates viral entry. However, owing to the rapid mutation of the influenza virus, which leads to antigenic drift, the HA antigen component of the mRNA vaccine requires annual review and modification. This feature makes the mRNA vaccine the most suitable platform for preventing influenza virus infection and controlling the spread of the disease. In 2012, Petsch et al. made a significant breakthrough by demonstrating the effectiveness of an mRNA vaccine against influenza encoding the full-length HA of influenza A/Puerto Rico/8/1934 (PR8HA).^116^ The serum of the mRNA-vaccinated mice showed effective seroconversion with an increased amount of virus-neutralizing antibodies. Moreover, the CD8^+^ T cells from the vaccinated mice had increased cytotoxic activity associated with viral clearance and long-term immunological memory. The administration of mRNA vaccines also induced long-term immunity and protected animals (mice, ferrets, and pigs) from influenza A virus infection.^116^ Of note, the mRNA vaccine encoding HA from the PR8 H1N1 strain triggered homologous and heterologous immune responses against H1N1 and H5N1 strains, suggesting protection against heterogeneous viruses.^116^ In 2017, Lutz et al. developed an LNP-enclosed mRNA vaccine encoding the HA of the influenza virus strain H1N1pdm09.^117^ The use of an mRNA vaccine induced an enhanced adaptive immune response represented by a transient local immunostimulatory milieu. The serum of the vaccinated mice showed an increased amount of multifunctional CD4^+^ and CD8^+^ T cells specifically against the influenza virus. The injection of the mRNA vaccine induced a stable humoral response against the influenza virus for at least one year, comparable with that of other inactivated virus-based licensed vaccines, as demonstrated by a continuous follow-up of functional antibody and T-cell responses.^117^ The same year, Bahl et al. developed mRNA vaccines encoding the HA proteins of H10N8 and H7N9,^118^ which induced robust humoral and cellular responses in preclinical mouse models, protecting mice from a lethal infection.^118^ Feldman et al. further performed the first randomized phase I clinical trial utilizing two mRNA vaccines against H10N8 and H7N9.^119^ These two vaccines were well tolerated without any serious vaccine-related adverse events. The HA inhibition titers after the intradermal administration of 50 μg mRNA vaccine were ≥1:40 in 89.7% of patients. However, a significantly enhanced cellular response was not detected.^119^ In 2021, Chivukula et al. used unmodified and LNP-encapsulated mRNA encoding full-length HA or full-length neuraminidase (NA).^120^ The HA and NA mRNA-LNP formulations, whether administered as monovalent or multivalent vaccines, have demonstrated the ability to elicit robust functional antibody and cellular immune responses in nonhuman primates. The induced antigen-specific antibody responses have been found to be correlated with protective effectiveness against viral challenge in mice. In 2022, McMahon et al. assessed immunogenicity and protective efficacy of a quadrivalent nucleoside-modified mRNA vaccine against influenza in mice. This vaccine formulation included four antigens from influenza A group 2 viruses: HA stalk, NA, matrix protein 2, and nucleoprotein.^121^ The vaccination elicited antigen-specific cellular and humoral immunity, protected mice from all challenge viruses, and provided protection from morbidity at a dose of 125 ng per antigen. The same year, Pardi et al. developed a pentavalent nucleoside-modified mRNA vaccine that offered broad protection against influenza B viruses encoding antigens, B/Yamagata/16/1988-like lineage HA, B/Victoria/2/1987-like lineage HA, NA, matrix-2, and nucleoprotein.^122^ This vaccine provided protection from morbidity at an impressively low dose of 50 ng per antigen. Additionally, Arevalo et al. developed a multivalent nucleoside-modified mRNA vaccine targeting all known influenza virus subtypes.^123^ This multivalent vaccine, which encoded HA antigens from all 20 known subtypes of influenza A/B virus lineages, elicited strong antibody responses in mice and ferrets. These antibodies showed reactivity against all 20 encoded antigens and provided protection to mice and ferrets when challenged with both matched and mismatched viral strains. In general, mRNA vaccines with a rapid speed of production may become a critical treatment against influenza viruses. Further randomized studies are necessary to confirm the safety and effectiveness of mRNA influenza vaccines.

### mRNA vaccines against cytomegalovirus

Human cytomegalovirus (CMV) belongs to the Betaherpesvirinae subfamily and possesses a genome size of 236 kilobases.^124^ Following primary infection, CMV typically establishes a latent state, persisting in the host without causing active disease. Virus reactivation in immunocompromised individuals can cause life-threatening complications involving the lung, gastrointestinal tract, liver, eye, or central nervous system. CMV is recognized as the most prevalent infectious cause of congenital malformations, with sensorineural hearing loss, developmental delay, and fetal death in 10–15% of cases.^124,125^ The process of viral entry into host cells is facilitated by the presence of viral envelope glycoproteins (g) gB and gH/gL (pentameric complex (PC)), and cell−cell fusion events allow the dissemination of the virus.^126,127^ In 2018, John et al. developed an mRNA vaccine encoding multiple CMV antigens, and the results using in vitro cell experiments showed that the mRNA-transfected cells expressed high levels of the encoded antigens. The administration of mRNA CMV vaccines in mice generated long-lasting and high titers of neutralizing antibodies against gB and PC. In addition, an enhanced proportion of IFN-γ-producing T cells was observed in vaccinated mice.^128^ In 2020, Nelson et al. tested an mRNA vaccine encoding full-length gB in rabbits, which showed enhanced virus neutralization ability and superior whole-virion phagocytosis activity compared with other vaccinated groups. The long-lasting immune response encourages the use of this mRNA vaccine in future clinical studies.^129^ In 2021, Webster et al. administered an mRNA vaccine encoding CMV gB and PC by intramuscular injection to cynomolgus and rhesus macaques, and an increased level of antigen-specific plasma antibody was detected in both species. The elicited antibodies against PC were dose dependent, while the boosted antibodies against gB were similar in groups treated with 20 μg vaccine and 120 μg vaccine. However, mRNA had no significant influence on antibody-induced cellular phagocytosis against CMV.^130^ Two phase I clinical trials are active but not recruiting to assess the reactogenicity, safety, and immunogenicity of the mRNA-1647 CMV vaccine (NCT05105048 and NCT05397223). A phase II clinical trial is recruited to assess the efficacy, safety, and immunogenicity of the mRNA-1647 CMV vaccine (NCT05683457). A phase I/II clinical study is also recruiting to assess the safety and immunogenicity of the mRNA-1647 CMV vaccine in healthy individuals 9 to 15 years of age and individuals 16 to 25 years of age (NCT05575492). A phase III clinical study is recruiting healthy participants 16 to 40 years of age to assess the efficacy, safety, and immunogenicity of the mRNA-1647 CMV vaccine (NCT05085366). A phase I trial evaluating the safety, reactogenicity as well as immunogenicity of mRNA-1647 and mRNA-1443 CMV vaccines have been completed in healthy adults, but the findings are not disclosed (NCT03382405). A dose-finding study to assess the safety and immunogenicity of CMV vaccine mRNA-1647 has also been completed in healthy adults, but the results are not reported (NCT04232280). Together, no clinical data have been reported regarding the safety, reactogenicity, safety, and immunogenicity of CMV mRNA vaccines to date. The publication of these data has the potential to offer significant insights for the advancement of anti-CMV mRNA vaccines.

### mRNA vaccines against respiratory syncytial virus

Respiratory syncytial virus (RSV) is an enveloped virus belonging to the Pneumovirus genus within the Paramyxoviridae family.^131,132^ It is the most common pathogen in infants and young children causing acute lower respiratory infection. Older adults, especially those with deficient immunity, are also susceptible to RSV. The fusion protein (F protein) is targeted by the human immune system against RSV; thus, it is usually selected as the antigen for vaccine development. However, when RSV attaches to the targeted cell, the F protein is modified in a prefusion form, which hides the potent neutralizing epitopes, leading to the immune evasion of RSV. In 2020, Espeseth et al. tested mRNA vaccines encoding RSV F proteins with different conformations, and the results demonstrated that the native form of RSV F protein generated high titers of neutralizing antibodies against both prefusion- and postfusion-specific epitopes.^133^ The mRNA vaccine encoding the F protein with prefusion stabilizing mutations can generate a humoral response toward prefusion-specific epitopes. However, the stabilizing mutations do not generate higher titers of neutralizing antibody or enhanced T-cell response compared with the effect of the mRNA vaccine encoding the native F protein.^133^ A phase I study is recruiting individuals aged 5 months to <24 months to evaluate the safety and immunogenicity of mRNA-1365 and mRNA-1345 (NCT05743881). A phase I trial is currently in progress, focusing on the tolerability and reactogenicity of mRNA-1345 in various populations (NCT04528719). This includes younger adults, women of child-bearing potential, older adults, and RSV-seropositive children. The study involves different dosing regimens, including ingle injections of up to 5 dose levels in younger adults, 3 injections of the middle dose level administered with a 56-day interval in younger adults, a booster injection around 12 months following the primary injection in older adults, and 3 injections of 1 of 2 dose levels given 56 days apart in RSV-seropositive children. Although infants and young children are frequently infected by RSV, few clinical trials have been performed at this stage to date, but they have been launched for adults. Moderna developed an mRNA vaccine named mRNA-1777 that encodes RSV F protein stabilized in the prefusion conformation, which became the first RSV mRNA vaccine entering a phase I clinical study for assessing its safety, tolerability, and immunogenicity.^134^ A total of 72 healthy young adults from 18 to 49 years old and 107 healthy old adults from 60 to 79 years old were enrolled in this study, randomly divided into two groups and treated with mRNA-1777 or placebo. The safety profile of mRNA-1777 was favorable, with no reports of serious adverse events and good tolerability observed. mRNA-1777 induced geometric mean titers of neutralizing antibody peaking from day 29 to 60 postinjection and declining over time. Intracellular cytokine staining of IFN-γ, IL-2, and TNF-α also showed enhanced CD4^+^ T-cell responses in both young and old participants. These results are promising for use in large randomized, placebo-controlled trials involving vulnerable adult populations in the future.^134^ In addition, multiple clinical trials have been performed. A phase I study is recruiting adults 50 to 75 years old for assessing the safety, reactogenicity, and immunogenicity of the mRNA-1045 RSV vaccine (NCT05585632). An observational study is currently recruiting participants to assess the real-world efficacy of the Moderna mRNA-1345 vaccine in preventing lower respiratory tract disease caused by RSV, as well as to investigate additional health and economic outcomes (NCT05572658). A phase I/II study is currently underway to evaluate the safety and immunogenicity of a single intramuscular injection of 3 dose levels of an RSV mRNA vaccine candidate formulated with two different LNPs (i.e., LNP containing CL-0059 or CL-0137) in healthy adults aged 18–50 years and 60 years and older (NCT05639894). A phase II/III study is recruiting adults aged 60 years and older to assess the safety and tolerability of the mRNA-1345 vaccine and the vaccine’s ability to prevent the first episode of RSV-associated lower respiratory tract disease in this population (NCT05127434). Although multiple clinical studies have been launched, almost all are still at an early stage, and the prophylactic effects of the mRNA vaccine against acute infection of the lower respiratory tract remain to be defined.

### mRNA vaccines against varicella-zoster virus

Varicella zoster virus (VZV), also referred to as human herpesvirus 3, is an alphaherpesvirus with a double-stranded DNA genome that is widely distributed in the human population.^102^ Primary VZV infection leads to varicella (chickenpox), and it becomes latent in ganglionic neurons. Latent VZV is reactivated in severe cases due to decreased cellular immunity against VZV, causing postherpetic neuralgia, which may lead to unbearable pain lasting for months and affect the quality of life of patients. VZV encodes 10 glycoproteins: ORFS/L, gK, gN, gC, gB, gH, gM, gL, gI and gE.^135–137^ In 2020, Monslow et al. developed an LNP-enclosed mRNA vaccine encoding the VZV gE antigen, and its efficacy was compared with that of two other vaccines approved on the market, including one with a live attenuated virus and one with a subunit protein. Rhesus monkeys were divided into five groups and treated with VZV gE subunit protein, live-attenuated VZV, and mRNA VZV vaccine at different doses. The results revealed the safety of the two 50 μg mRNA VZV vaccines and the ability to trigger a potent humoral and cellular immunity comparable to that of the 50 μg subunit protein vaccine, indicating that the mRNA vaccine is a suitable platform for future production of the VZV vaccine.^138^ Although the translatability of the results was promising, more clinical and preclinical investigations focused on the effectiveness and safety of the vaccine are still urgently needed.

### mRNA vaccines against rabies virus

Rabies virus is a negative-stranded RNA virus of the Rhabdoviridae family causing rabies, a zoonotic viral disease with nearly 100% fatality.^139^ The rabies virus binds to its cellular target through the surface glycoprotein RABV-G, gaining access to the peripheral nerves and the central nervous system. In 2016, Schnee et al. tested a vaccine composed of mRNA encoding RABV-G in mice and domestic pigs and discovered 2 doses of this vaccine-induced virus-specific neutralizing titers ≥0.5 IU/ml and an increased proportion of virus-specific CD4^+^ T cells.^65^ Antibody titers in mice vaccinated with 20 μg and 80 μg mRNA vaccine remained stable throughout one year of measurement once a month, with mean titers of approximately 40 IU/ml. The vaccinated mice were protected against intracerebral rabies virus infection, suggesting the satisfying immunogenicity of the mRNA vaccine.^65^ In 2017, Alberer et al. performed the first phase I clinical study in Germany using the mRNA rabies vaccine CV7201.^140^ A total of 101 participants aged 18 to 40 were enrolled and vaccinated, and the results demonstrated that CV7201 was generally safe and well tolerated, with only one vaccine-related serious side effects (moderate Bell’s palsy). RABV-G-specific IgM and IgG titers peaked at days 21 and 42 postinjection. A significant enhancement in serum IgG was found after the 1-year boost, suggesting the establishment of an immune memory response in participants. RABV-G-specific CD4^+^ T cells transiently enhanced after vaccination and declined to baseline levels 3 months after injection.^140^ Since the phase I clinical trial using mRNA rabies vaccine showed satisfying outcomes, future studies should focus on increasing antibody titers inducing a longer immune response to potentially help the production of cheaper and more available rabies vaccines to meet the needs of public health.

## mRNA vaccines in cancers

**Fig. 4:**
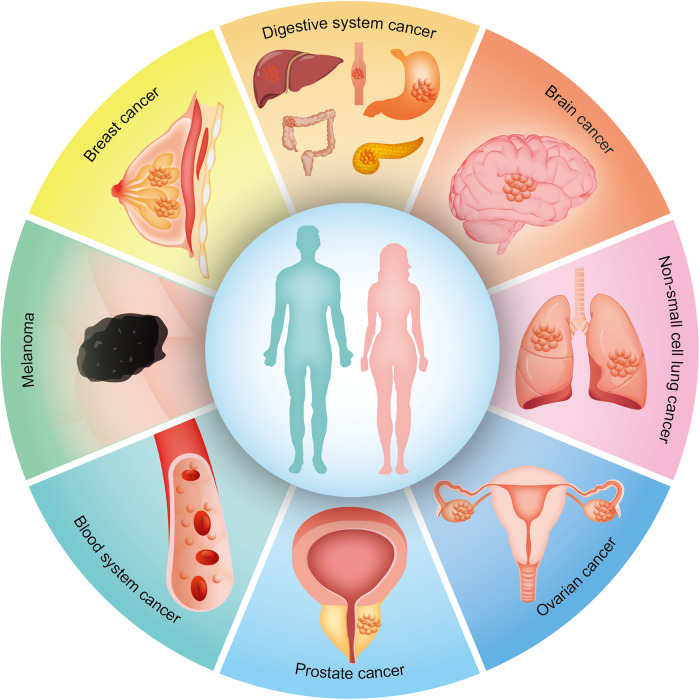
Landscape of mRNA vaccines in cancers. mRNA vaccines have been developed against multiple cancers to date, including melanoma, brain cancer, non-small cell lung cancer, ovarian cancer, prostate cancer, blood system cancer, digestive system cancer, and breast cancer. This figure is created using Adobe Illustrator and integrates the current literature-based knowledge

mRNA vaccines in cancers are usually applied in a therapeutic setting instead of a prophylactic approach in infectious diseases.^141^ Indeed, it is typically designed to encode tumor-associated antigens (TAAs) or neoantigens to activate antitumour immune responses.^142^ To date, numerous clinical trials investigating the effect of the mRNA vaccine against various cancers have been registered in the U.S. National Library of Medicine (ClinicalTrials.gov), including melanoma, brain cancer, non-small cell lung cancer (NSCLC), ovarian cancer, prostate cancer, blood system cancer, digestive system cancer, and breast cancer (Table 2 and Fig. 4).

**Table 2:** Clinical trials for mRNA vaccines in cancers

Disease	NCT Number	Disease condition	Encoded antigen	Vehicle	Combination	Phase	Status
Melanoma	NCT00204516	Stage III/IV	Melan-A, MAGE-A1, MAGE-A3, survivin, gp100, tyrosinase, personalized antigens	Unknown	GM-CSF	Phase I/II	Completed
	NCT00204607	Stage III/IV	Melan-A, MAGE-A1, MAGE-A3, survivin, gp100, tyrosinase	Protamine	GM-CSF	Phase I/II	Completed
	NCT01278940	Advanced	Tumor mRNA-encoded antigens	DC	IL-2	Phase I/II	Completed
	NCT01530698	Stage III/IV	GP100, tyrosinase	DC	No	Phase I/II	Completed
	NCT01676779	Stage III/IV	Unknown	DC	No	Phase II	Completed
	NCT00243529	Stage III/IV	GP100 and tyrosinase	DC	No	Phase I/II	Completed
	NCT01066390	Stage III/IV	MAGE-A3, MAGE-C2, tyrosinase, gp100	DC	No	Phase I	Completed
	NCT00978913	Unknown	Survivin, hTERT, p53	DC	Cyclophosphamid	Phase I	Completed
	NCT00940004	Stage III/IV	GP100, tyrosinase	DC	No	Phase I/II	Completed
	NCT02285413	Stage III/IV	GP100, tyrosinase	DC	Cisplatinum	Phase II	Completed
	NCT00672542	Metastatic	Melan-A, tyrosinase, gp100, MAGE-3	DC	Proteasome siRNA-tranfected DC	Phase I	Completed
	NCT01278940	Advanced	Autologous tumor mRNA-encoded antigens	DC	IL-2	Phase I/II	Completed
	NCT01684241	Advanced	NY-ESO-1, tyrosinase	No	No	Phase I	Completed
	NCT04526899	Anti-PD-1-refractory/relapsed, unresectable stage III or IV	MAGE-A3, NY-ESO-1, TPTE, tyrosinase	Liposome	Cemiplimab	Phase II	Recruiting
	NCT01983748	Uveal	Autologous tumor RNA-encoded antigens	DC	No	Phase III	Recruiting
	NCT03739931	Advanced	OX40L, IL-23, IL-36γ	Lipid Nanoparticle	Durvalumab	Phase I	Recruiting
	NCT03897881	Unknown	Personalized antigens	Lipid Nanoparticle	Pembrolizumab	Phase II	Active, not recruiting
	NCT01456104	Stage II/III/IV	Murine tyrosinase-related peptide 2	DC	No	Phase I	Active, not recruiting
	NCT02410733	Stage III/IV	NY-ESO-1, MAGE-A3, TPTE, tyrosinase	Liposome	Pembrolizumab	Phase I	Active, not recruiting
	NCT04335890	Metastatic uveal	Autologous tumor-RNA-encoded antigens	DC	No	Phase I	Active, not recruiting
	NCT00126685	Stage IV	Autologous polymerase chain reaction-amplified tumor RNA-encoded antigens	DC	No	Phase I/II	Unknown
	NCT00929019	Unknown	GP100, tyrosinase	DC	No	Phase I/II	Terminated
	NCT00961844	Stage II/III/IV	hTERT, survivin, tumor cell-derived mRNA-encoded antigens	DC	Temozolomid	Phase I/II	Terminated
	NCT03394937	Unknown	5 TAAS	No	Pembrolizumab	Phase I	Terminated
	NCT03480152	Metastatic	Autologous cancer cell-derived neoantigens	Unknown	No	Phase I/II	Terminated
Brain Cancer	NCT00846456	Glioblastoma	Glioblastomas stem cell-derived mRNA-encoded antigens	DC	No	Phase I/II	Completed
	NCT00890032	Recurrent glioblastoma multiforme	Autologous brain tumor stem cell mRNA-encoded antigens	DC	No	Phase I	Completed
	NCT00626483	Glioblastoma multiforme	CMV pp65 LAMP	DC	Basiliximab, GM-CSF	Phase I	Completed
	NCT02529072	Recurrent astrocytoma, malignant glioma, and glioblastoma	CMV pp65 LAMP	DC	Nivolumab	Phase I	Completed
	NCT03615404	Glioblastoma, malignant glioma, medulloblastoma recurrent, pediatric glioblastoma multiforme, pediatric brain tumor, recurrent pediatric brain tumor	CMV pp65 LAMP, GM-CSF	DC	Tetanus toxoid	Phase I	Completed
	NCT04963413	Glioblastoma	CMV pp65 LAMP, GM-CSF	DC	No	Phase I	Recruiting
	NCT04573140	Adult glioblastoma, pediatric high-grade gliomas	Autologous total tumor mRNA, CMV pp65 LAMP	DOTAP liposome	No	Phase I	Recruiting
	NCT03548571	IDH wild-type, MGMT-promotor methylated glioblastoma	Survivin, hTERT, autologous tumor stem cell mRNA-encoded antigens	DC	Temozolomide	Phase II/III	Recruiting
	NCT02649582	Glioblastoma multiforme	WT1	DC	Temozolomide	Phase I/II	Recruiting
	NCT02465268	Glioblastoma multiforme	CMV pp65 LAMP	DC	Saline, Td, GM-CSF	Phase II	Recruiting
	NCT03688178	Glioblastoma	CMV pp65 LAMP	DC	Varlilumab	Phase II	Recruiting
	NCT00639639	Glioblastoma multiforme	CMV pp65 LAMP	DC	Tetanus-Diphtheria, Toxoid	Phase I	Active, not recruiting
	NCT03927222	Glioblastoma	CMV pp65 LAMP	DC	Temozolomide, Tetanus-Diphtheria Toxoid, GM-CSF	Phase II	Suspended
	NCT04741984	Glioblastoma	CMV pp65 LAMP	Monocytes	No	Phase I	Not yet recruiting
	NCT02808364	Recurrent glioblastoma	Personalized antigens	DC	No	Phase I	Unknown
	NCT02709616	Glioblastoma	Personalized antigens	DC	Temozolomide, Radiotherapy	Phase I	Unknown
	NCT02366728	Glioblastoma	CMV pp65 LAMP	DC	Basiliximab, Temozolomide, Saline	Phase II	Unknown
	NCT01291420	Glioblastoma	WT1	DC	No	Phase I/II	Unknown
Non-Small Cell Cancer	NCT03164772	Metastatic	NY-ESO-1, MAGE-C1, MAGE-C2, 5T4, survivin, muclin-1	No	Durvalumab, Tremelimumab	Phase I/II	Completed
	NCT00923312	Stage IIIB/IV	NY-ESO-1, MAGE-C1, MAGE-C2, 5T4, survivin	No	No	Phase I/II	Completed
	NCT03948763	KRAS Mutant advanced or metastatic	KRAS	Lipid Nanoparticle	Pembrolizumab	Phase I	Recruiting
	NCT03908671	Stage IIIB/IV	Personalized neoantigens	Unknown	No	Not applicable	Not yet recruiting
	NCT03908671	Unresectable or metastatic	Personalized neoantigens	Unknown	No	Not applicable	Unknown
Ovarian Cancer	NCT04163094	Primary	3 OC TAAs	Liposome	Carboplatin/Paclitaxel, Surgery	Phase I	Active, not recruiting
	NCT03323398	Advanced or metastatic	OX40L	Lipid Nanoparticle	Durvalumab	Phase I/II	Active, not recruiting
	NCT01334047	Relapsed	hTERT, survivin, stem cell mRNA-encoded antigens	DC	No	Phase I/II	Terminated
	NCT01456065	Stage III	hTERT	DC	Survivin	Phase I	Unknown
Prostate Cancer	NCT01278914	Androgen resistant metastatic	Unknown	DC	Unknown	Phase I/II	Completed
	NCT01446731	Castration-resistant metastatic	PSA, PAP, survivin, hTERT	DC	Docetaxel	Phase II	Completed
	NCT00831467	Hormone refractory	PSA, PSMA, PSCA, STEAP	Unknown	No	Phase I/II	Completed
	NCT00004211	Metastatic	PSA	DC	No	Phase I/II	Completed
	NCT00010127	Metastatic	Autologous tumor mRNA-encoded antigens	DC	No	Phase I	Terminated
	NCT04382898	Metastatic castration-resistant or high-risk localized	RBL038, RBL039, RBL-040, RBL-041, RBL-045	Liposome	Cemiplimab	Phase I/II	Active, not recruiting
	NCT01197625	High Gleason score (9–10) or micrometastatic	Primary prostate cancer tissue mRNA-encoded antigens	DC	hTERT, Survivin	Phase I/II	Active, not recruiting
	NCT02140138	Intermediate to high risk	PSA, PSMA, PSCA, STEAP, PAP, mucin-1	DC	No	Phase II	Terminated
	NCT01153113	Metastatic	hTERT	DC	No	Phase I/II	Withdrawn
	NCT00006430	Metastatic	Autologous prostate tumor tissue mRNA-encoded antigens	DC	No	Phase I	Unknown
Blood System Cancer	NCT00834002	AML	WT1	DC	No	Phase I	Completed
	NCT01734304	AML	WT1, PRAME, CMV pp65 LAMP	DC	No	Phase I/II	Completed
	NCT00510133	AML	hTERT, LAMP	DC	No	Phase II	Completed
	NCT01686334	AML	WT1	DC	No	Phase I/II	Recruiting
	NCT03083054	AML, high risk myelodysplastic syndromes	WT1	DC	No	Phase I/II	Active, not recruiting
	NCT00514189	AML	AML mRNA-encoded antigens	DC	No	Phase I	Terminated
	NCT00965224	AML, CML, myeloma	WT1	DC	No	Phase II	Unknown
	NCT02315118	CD20 + CLL, B-cell non-Hodgkin’s lymphoma	CD16-41BB-CD3zeta	T cell	Rituximab, IL-2	Phase I/II	Unknown
	NCT03739931	Lymphoma	OX40L, IL-23, IL-36γ	Lipid Nanoparticle	Durvalumab	Phase I	Recruiting
	NCT03323398	Lymphoma	OX40L	Lipid Nanoparticle	Durvalumab	Phase I/II	Active, not recruiting
	NCT01995708	Multiple myeloma	CT7, MAGE-A3, WT1	DC	Autologous stem cell transplantation	Phase I	Active, not recruiting
Digestive System Cancer	NCT00228189	CRC with liver metastases	CEA	DC	No	Phase I/II	Completed
	NCT03480152	Metastatic gastrointestinal cancer	Personalized neoantigens	Lipid nanoparticle	No	Phase I/II	Completed
	NCT00003433	Metastatic CRC	CEA	DC	No	Phase I/II	Completed
	NCT03468244	Advanced ESC, GA, PAAD, CRC	Personalized neoantigens	Unknown	No	Not applicable	Recruiting
	NCT03948763	KRAS mutant advanced or metastatic CRC, PAAD	KRAS	Lipid Nanoparticle	Pembrolizumab	Phase I	Recruiting
	NCT03431311	Metastatic CRC	TGF-β receptor type II	T cell	No	Phase I/II	Terminated
	NCT02693236	Middle and advanced esophagus cancer	Mucin-1, survivin	DC	Cytokine-induced killer cells	Phase I/II	Unknown
	NCT03908671	Advanced esophagus cancer	Personalized neoantigens	Unknown	No	Not applicable	Not yet recruiting
	NCT03480152	HCC, colon cancer, gastrointestinal cancer	Personalized neoantigens	Unknown	No	Phase I/II	Terminated
	NCT03908671	Advanced esophageal cancer	Personalized neoantigens	Unknown	No	Not applicable	Unknown
Breast Cancer	NCT00978913	Metastatic	Survivin, hTERT, p53	DC	Cyclophosphamide	Phase I	Completed
	NCT03788083	Early	TriMix	No	Placebo	Phase I	Recruiting
	NCT03739931	Triple negative	OX40L, IL-23, IL-36γ	Lipid Nanoparticle	Durvalumab	Phase I	Recruiting
	NCT02316457	Triple negative	Personalized tumor antigens	Unknown	No	Phase I	Active, not recruiting
	NCT01291420	Metastatic or locally advanced	WT1	DC	No	Phase I/II	Unknown
	NCT00003432	Metastatic	CEA	DC	No	Phase I/II	Terminated

*AML* acute myelocytic leukemia, *CML* chronic myeloid leukemia, *CLL* chronic lymphocytic leukemia, *CRC* colorectal cancer, *HCC* hepatocellular carcinoma, *ESC* esophageal squamous carcinoma, *GA* gastric adenocarcinoma, *PAAD* pancreatic adenocarcinoma, *TAAs* tumor-associated antigens, *TriMix* mRNA encoding TLR4, CD40L, and CD70, *DC* dendritic cell

### mRNA vaccines against melanoma

Melanoma arises from the malignant conversion of melanocytes that are widely distributed in the body (e.g., skin, mucosa, uvea, inner ear, and rectum). Cutaneous melanoma is the most common type that accounts for ~1.7% of all newly diagnosed primary malignancies, responsible for ~0.7% of all cancer mortality worldwide.^143^ DC-based mRNA vaccines are mostly used to combat melanoma. As early as 1996, Boczkowski et al. performed adoptive mRNA-pulsed DC transfer, discovering that a DC-pulsed mRNA vaccine encoding ovalbumin (OVA) protected mice from OVA-expressing tumor cells and significantly reduced lung metastases in a B16/F10.9 tumor model.^144^ In recent years, diverse DC-based mRNA vaccines have been tested in melanoma patients. Gaudernack et al. isolated autologous total mRNA from biopsied melanoma tissue and introduced it into DCs via electroporation.^145,146^ Then, melanoma patients were vaccinated with autologously derived tumor mRNA-transfected DCs, causing the induction of a wide range of T-cell responses. Several antigens have been used as targets for mRNA vaccine development, such as MAGE-A3, MAGE-A2, gp100, and tyrosinase. MAGE-A3 and MAGE-C2 are exclusively expressed in germ cells and tumor cells (including melanoma cells), while tyrosinase and gp100 are widely expressed in both tumor and normal tissue. Aarntzen et al. utilized mRNA to electroporate monocyte-derived DCs to encode gp100 and tyrosinase.^147,148^ These monocyte-derived DCs were then administered to 45 patients with stage III and IV melanoma. The study demonstrated notable CD4^+^ and CD8^+^ T-cell responses specific to tumor antigens, suggesting the potential effectiveness of mRNA-electroporated DC vaccines for treating melanoma. Immunological adjuvants are usually used to stimulate and amplify the immune responses to the targeted antigens to regulate the in vivo immunogenicity of the mRNA vaccine. TriMix has been mostly used as a DC-based mRNA vaccine against melanoma because it encodes the activation stimulator CD40L (CD4^+^ T-cell activator), the costimulatory molecule CD70 (CD8^+^ T-cell activator), and the constitutively active TLR 4 (DC antigen presentation promotor). Wilgenhof et al. described a phase IB trial that enrolled 15 advanced melanoma patients who were subjected to vaccination with autologous monocyte-derived DCs electroporated with synthetic mRNA (TriMixDC-MEL).^149^ The report revealed that the mRNA encoded CD40L, TLR4, CD70, HLA-II targeting signal, and a TAA (MAGE-A3, MAGE-C2, tyrosinase, or gp100). After vaccination, two patients exhibited a complete response, two patients exhibited a partial response, and all patients showing an objective response had a progression-free disease > two years. In addition, Jansen et al. showed a phase II trial using TriMixDC-MEL as an adjuvant treatment in stage III/IV melanoma.^150^ The findings of the study arm displayed 71% of patients were free of disease as compared to 35% in the control arm one year later. Given that the expression of PD-1/PD-L1 can compromise the efficacy activated by mRNA vaccines, one study investigated the effect of combined treatment of TriMixDC-MEL and an immune checkpoint inhibitor (ipilimumab) in stage III/IV melanoma (NCT01302496).^151^ The treatment elicited objective long-term clinical responses, with an overall survival of 28% (11/39) and progression-free survival of 18% (7/39) after 5 years of follow-up. Eighty percent (12/15) of patients with immune monitoring were vaccine responders, and among them, 10 showed T-cell responses against at least two antigens.

Although in vitro transcription-based forms are less common than DC-based forms, the development in molecular biotechnology has made this form feasible for cancer treatment. BioNTech, GenenTech, and Moderna are leading companies in the pharmaceutical industry, and as such, they have announced clinical updates regarding in vitro transcription-based vaccines against melanoma. In 2004, Weide et al. performed a phase I/II study to assess the safety and efficacy of a protamine-mRNA vaccine encoding TAAs (gp100, Melan-A, Tyrosinase, MAGE-A1, MAGE-A3, and survivin) in a group of 21 patients with metastatic melanoma.^152^ After vaccination, one patient among 7 with measurable disease experienced a complete response. Foxp3^+^/CD4^+^ regulatory T cells or myeloid suppressor cells were significantly reduced in the peripheral blood of vaccinated patients with or without keyhole limpet hemocyanin, respectively. Two of 4 immunologically evaluable patients displayed a reproductive elevation in vaccine-specific T cells. BNT111 is a liposomal RNA vaccine encoding four TAAs, MAGE-A3, NY-ESO-1, PTEN, and tyrosinase, and its safety and effectiveness were evaluated in 2015 after intravenous administration in a phase I trial (Lipo-MERIT, NCT02410733).^153^ This study recruited 89 patients, with 42 suffering from measurable stage III/IV melanoma. Three patients in the BNT111 monotherapy group exhibited a partial response, seven exhibited stable disease, and one exhibited complete metabolic remission of metastatic lesions, as revealed by PET/CT imaging. The combination of BNT111 with PD-1 blockade revealed that six of 17 patients experienced a partial response. The disease was controlled for a long time in most of the patients with partial response or stable disease in both groups during a follow-up period of up to two years. The observed clinical response was accompanied by the activation of CD4^+^ and CD8^+^ T-cell immune responses specifically targeting the vaccine antigens. Additionally, the therapeutic adverse events experienced by patients were predominantly mild to moderate flu-like symptoms such as fever and chills. These symptoms were mostly observed early on, of short duration, easily manageable, and typically resolved within 24 h. At present, BNT111 is being used in an ongoing phase II trial for the treatment of PD-1 inhibitor refractory/recurrent and unresectable stage III/IV melanoma (NCT04526899). On November 19, 2021, BioNTech was granted priority eligibility for the treatment of melanoma with BNT111 by the FDA. In 2022, Sittplangkoon et al. studied the immunogenicity and antitumour responses of mRNA that encodes tumor antigens with varying levels of N1-methylpseudouridine modification in a B16 melanoma model.^154^ The mRNA vaccine encoding OVA-induced significant production of IFN-I and the maturation of DCs, with a negative correlation observed with elevating percentages of N1-methylpseudouridin modification. Unmodified OVA-LNPs significantly reduced tumor growth, prolonged survival, and increased intratumoural CD40^+^ DCs and the frequency of granzyme B^+^/IFN-g^+^/TNF-a^+^ polyfunctional OVA peptide-specific CD8^+^ T cells in a B16-OVA murine melanoma model. The robust antitumour effects of unmodified OVA-LNPs were also found in the lung metastatic tumor model. In addition, the mRNA vaccine was also evaluated using B16 melanoma neoantigens (Pbk-Actn4), leading to a delay in tumor growth. Additionally, in 2017, Fernandez et al. launched a phase I trial to evaluate the immunogenicity and safety of the ECI006 vaccine in melanoma (a combination of TriMix and TAA-encoding mRNA) (NCT03394937). Nevertheless, the abovementioned mRNA vaccines are designed to target TAAs, and central tolerance is inevitable. Therefore, personalized mRNA vaccines are warranted to overcome this challenge.

The initial application of personalized RNA mutanome vaccines in human melanoma was reported in 2017.^155^ The authors identified nonsynonymous mutations in 13 melanoma patients by RNA and exome sequencing, and among them, ten per patient were selected to construct two synthetic RNAs according to the affinity to HLA class I/II. All patients were treated with a minimum of eight and a maximum of 20 neoepitope vaccine injections. Increased responses were observed in one-third of patients who previously showed weak responses against neoepitopes, while de novo responses were observed in the remaining patients. Eight patients with no radiologically detectable lesions at the beginning of the vaccination generated a vigorous immune response and showed progression-free disease for 12–23 months. Moreover, vaccination induced a significant decrease in the cumulative rate of metastatic events and sustained progression-free survival. When these RNA mutanome vaccines were used in combination with PD-1 blockade, a third of patients experienced a complete response to the vaccination. The study revealed that the vaccination was well tolerated, with seven patients showing vaccine-related immune responses. Apart from this trial, another phase I multicenter study tested mRNA-4157 (a lipid-encapsulated personalized vaccine that encodes neoantigens selected based on a proprietary algorithm) monotherapy or combined with pembrolizumab in resected solid tumors (including melanoma).^147^ Among the 13 patients in the monotherapy arm that included three suffering from melanoma, 12 remained disease-free after a median follow-up of 8 months, and no drug-related adverse events of more than grade two were observed. Moreover, GenenTech and BioNTech started a series of phase I and II trials for personalized lipid-encapsulated mRNA vaccines combined with atezolizumab or pembrolizumab (e.g., NCT03289962 and NCT03815058).^147^ All these pieces of evidence demonstrate that personalized mRNA vaccines in combination with other anticancer approaches may pave the way for the treatment of melanoma.

Diverse mRNA vaccines have been developed for the treatment of melanoma, displaying potential therapeutic efficacy in clinical studies. However, no mRNA vaccine has been officially approved for the treatment of melanoma. The combination of mRNA vaccines with other therapeutic strategies may further enhance their effectiveness and promote their potential for approval.

### mRNA vaccines against brain cancer

Primary brain cancer is less frequent in adults, representing 1–2% of all cancer types worldwide.^156,157^ Malignant glioma is the most common subtype in brain cancer, with glioblastoma being the most aggressive subtype.^158^ The 5-year survival of brain cancer depends on its malignancy, with an approximate value of 32% in malignant glioma and approximately 5% in glioblastoma in the United States.^159^ DC-pulsed tumor mRNA vaccine is one of the first mRNA forms applied in human malignant glioma.^160^ Two studies involved the application of autologous tumor mRNA-loaded DCs. The first is a clinical study that recruited five patients who underwent subtotal removal of malignant glioma without receiving other therapy.^161^ All patients exhibited a specific CD8^+^ cytotoxic T-cell response after treatment with autologous tumor mRNA-loaded DCs, and among them, three showed potent cytolytic activity against autologous glioma cells. The other study recruited seven glioblastoma patients in a phase I/II study for evaluating the efficacy of DC-pulsed cancer stem cell mRNA.^162^ Two vaccinations were performed in all patients in the first week after the end of the standard chemoradiotherapy, followed by one weekly vaccination for 3 weeks and then one vaccination or temozolomide every 2 weeks. Although tumor recurrence was observed in five patients (at 10, 15, 17, 22, and 29 months after the treatment), six patients in the control group died before the first patient experiencing recurrence in the vaccinated group, and three of the seven survived for more than 1000 days. To exert more specific antitumour effects, a randomized and blinded clinical study on glioblastoma used a DC-pulsed mRNA vaccine encoding CMV pp65 since this protein is expressed in >90% of glioblastomas but not in the surrounding normal tissue.^163^ The authors assessed the impact of vaccine site preconditioning on DC migration. Twelve patients were randomly classified into two groups and subjected to unilateral vaccine site preconditioning with tetanus/diphtheria toxoid or unpulsed autologous DCs. Treatment with tetanus/diphtheria and mRNA vaccines significantly prolonged both overall and progression-free survival, and 50% of patients were alive for more than 36.6 months. A pp65-specific immune response was detected for several months in all the long-term survivors, and the increased pp65-specific interferon-γ levels were correlated with overall survival. A subsequent DC migration study involved 100 patients with resected, grade IV glioblastoma, but the results were not provided (NCT02366728). Two clinical trials are recruiting patients to investigate the effectiveness of human CMV pp65-LAMP in glioblastoma, and the results are not disclosed (NCT02465268, NCT03688178). In addition, a pp65-LAMP mRNA-loaded 1,2-dioleoyl-3-trimethylammonium-propane (DOTAP) liposome vaccine is being tested in high-grade glioma and glioblastoma in a phase I study, and the results are not published (NCT04573140). Of note, mRNA vaccines in malignant glioma mainly encode TAAs, and whether neoantigen-based mRNA vaccines together with immune checkpoint inhibitors could show superior efficacy in glioma remains to be investigated.

### mRNA vaccines against non-small cell lung cancer

Lung cancer is the second most frequent cancer and the leading cause of cancer mortality worldwide,^156^ with NSCLC representing 85% of all lung cancers.^164^ The 5-year survival rate is about 60% in cases of resectable diseases, approximately 33% in cases of unresectable regional disease, and 6.3% in cases of extended disease with metastasis.^164,165^ CV9201 and BI1361849 (CV9202) are two mRNA-based vaccines that were clinically tested in NSCLC.^166^ CV9201 is composed of a protamine-formulated sequence-optimized mRNA that encodes five NSCLC-associated antigens: MAGE-C1, MAGE-C2, NY-ESO-1, 5T4 and survivin. CV9202 has the same composition as CV9201 with the addition of mucin-1. Multiple clinical studies have been initiated to investigate their efficacy in NSCLC. In 2019, Sebastian et al. reported a phase I/IIA study using CV9201 in stage IIIB/IV NSCLC.^167^ A total of 46 locally advanced (*n* = 7) or metastatic (*n* = 39) NSCLC patients with stable disease after first-line treatment were recruited and subjected to five intradermal CV9201 injections (400–1600 µg of mRNA). The maximum dose was recommended in phase IIA, all doses were well tolerated, and most adverse events were mild-to-moderate reactions in the injection site and flu-like symptoms. An antigen-specific immunity was observed in 63% of assessable patients in phase IIA, and 60% (18/30) showed more than twofold activated IgD^+^CD38^hi^ B cells. A total of 31% (9/29) and 69% (20/29) of patients showed stable and progressive disease, respectively. The median overall and progression-free survival rates were 5 months and 10.8 months, respectively, and the 2- and 3-year survival rates were 26.7% and 20.7%, respectively. In the same year, Papachristofilou et al. reported a phase IB trial evaluating the effectiveness of CV9202 in combination with local radiation treatment in 26 patients suffering from stage IV NSCLC with stable disease or partial response after standard first-line treatment.^168^ These patients were classified into three strata: 1: no nonsquamous NSCLC, partial response/stable disease after treatment with four or more cycles of pemetrexed- and platinum-based therapy and no mutation of EGFR (*n* = 16); 2: squamous NSCLC, partial response/stable disease after four or more cycles of nonplatinum compound and platinum-based treatment (*n* = 8); and 3: nonsquamous NSCLC, stable disease/partial response after treatment for 3–6 months with EGFR-tyrosine kinase inhibitor, *EGFR* mutation (*n* = 2). Patients received two injections of CV9202, followed by radiation therapy (4 × 5 Gy). Patients in strata 1 and 3 subsequently were administrated with three further treatments with CV9202, while those in stratum 2 received four, after which all patients were vaccinated with CV9202 at 3-week intervals for the first 6 months and then every 6 weeks thereafter. Vaccination of CV9202 was continued until disease progression required systemic second-line treatment or in cases of patients encountering unacceptable toxicity. Patients in strata 1 and 3 received maintenance pemetrexed or continued EGFR-tyrosine kinase inhibitor therapy, respectively. An antigen-specific immune response was detected in all three strata (a total of 25 evaluable patients), and at least a twofold increase in the magnitude of the immune response against one or more of the CV9202 antigens compared to baseline was observed in 20 patients. Ten patients showed at least a twofold increase in functional CD8^+^/CD4^+^ T cells compared to the value at baseline. Twelve patients (12/26) exhibited stable disease, and one showed a partial response and was also treated with pemetrexed maintenance. The most common CV9202-related side effects were flu-like symptoms and reactions at the injection site, with three patients developing grade 3 (fatigue and pyrexia). Recently, a phase I/II study (NCT03164772) completed the assessment of the safety and effectiveness of CV9202 in combination with the immune checkpoint inhibitor durvalumab targeting PD-L1 and tremelimumab targeting CTLA-4 for the treatment of NSCLC, but the results are not published. In addition, a clinical study (NCT03908671) involving patients with NSCLC and advanced esophageal cancer for the use of a personalized mRNA vaccine that encodes tumor-specific antigens has been registered. Although a fraction of patients with NSCLC experience beneficial effects from treatment with the mRNA vaccine, the overall survival is still limited, as reported in published studies. Further optimization of the mRNA vaccine and the selection of suitable combination therapy is required to enhance its efficacy. In addition, only a few studies have been completed with published findings, and more clinical trials are needed for the future application of mRNA vaccines in NSCLC.

### mRNA vaccines against ovarian cancer

Ovarian cancer is one of the most dangerous gynecological cancers, with around 314 000 new cases and 207 000 mortalities in 2020 worldwide.^156^ It accounts for ~5% of female cancer-related death and has become the fifth leading cause of female cancer-related death global. A DC-pulsed mRNA vaccine encoding folate-receptor-α (FR-α) was used in 2004 for treating relapsed metastatic ovarian cancer.^169^ The patient involved in this study was a 62-year-old woman diagnosed with advanced serous papillary ovarian cancer IIIc with widespread peritoneal carcinomatosis and increased CA-125. The patient was subjected to two tumor debulking procedures and experienced two tumor relapses. The vaccine treatment with autologous DCs engineered with mRNA encoding FR-α started at the moment of the second relapse, with a total of ten vaccinations administered at 4-week intervals. The CT showed a partial response when the tumor volume was compared before the treatment and 3 months after the last vaccination. The CT at 16 months of follow-up revealed a regression of over 50% of the lymph-node metastases, and consistently, the vaccinations induced an FR-α-specific immune response. After six vaccinations, the IFN-γ produced by FR-α-stimulated CD8^+^ cells and FR-α-stimulated CD4^+^ cells increased 30-fold and 15-fold compared to the amount in the prevaccination samples, respectively. Similarly, granzyme B was increased after vaccination compared to the amount in the prevaccination samples. No systemic or local side reactions associated with the therapy were observed, indicating that the vaccination was well tolerated. Another publication reported the application of DC-pulsed mRNA encoding WT1 in ovarian carcinoma and carcinosarcoma.^170^ Two patients, one with serous ovarian cancer and the other with ovarian carcinosarcoma, received four weekly vaccinations, which induced increased CD137^+^ antigen-specific T cells, IL-2, and IFN-γ in ovarian carcinoma and CD137^+^ antigen-specific T cells, IL-2, and TNF-α in ovarian carcinosarcoma. Unfortunately, the disease progressed after four vaccinations, and the patient with ovarian carcinoma survived for 19 months, while the patient with ovarian carcinosarcoma survived for 12 months after the end of vaccine administration. In that same year, a phase I study was conducted to assess the safety of active immunotherapy using fully mature, TERT-mRNA, and survivin-peptide double-loaded DCs in 15 patients with advanced epithelial ovarian cancer. However, the results were not published despite the completion of the study (NCT01456065). A first-in-human, open-label phase I study is currently recruiting ovarian cancer patients for a liposome-formulated mRNA vaccine together with (neo)-adjuvant chemotherapy (NCT04163094). Regrettably, the phase I/II trial that utilized autologous DCs loaded with amplified ovarian cancer stem cell mRNA, hTERT, and survivin in recurrent platinum-sensitive epithelial ovarian cancer patients was terminated in 2021 (NCT01334047), with no results disclosed. The utilization of mRNA vaccines in ovarian cancer is still in its early stages, and the number of patients enrolled in clinical trials remains limited. The efficacy of mRNA vaccines in ovarian cancer should be further explored in a larger number of patients for a better evaluation of their efficacy.

### mRNA vaccines against prostate cancer

Prostate cancer is the second most common cancer and the fifth leading cause of cancer-related mortality among men globally, with approximately 10 million men diagnosed.^156,171^ It causes over 400,000 mortalities annually worldwide, which is projected to reach over 800,000 deaths annually by 2040.^156,171–173^ Islam et al. developed an adjuvant-pulsed mRNA vaccine nanoparticle containing an OVA-coded mRNA and a palmitic acid-modified TLR7/8 agonist R848 (C16-R848) encapsulated with a lipid-polyethylene glycol shell.^174^ This vaccine successfully preserved the adjuvant activity of the encapsulated C16-R848, and exhibited a notable improvement in mRNA transfection efficacy, with a rate exceeding 95%. This high transfection efficacy led to enhanced presentation of the OVA mRNA-derived antigen on MHC class I molecules in antigen-presenting cells. Vaccination elicited potent adaptive immune responses by improving the extension and infiltration of OVA-specific CD8^+^ T cells in OVA-expressing syngeneic allograft mouse models of prostate cancer and suppressed tumor growth when offered postengraftment (60% reduction vs. control). CV9103 encodes four TAAs in prostate cancer: PSA, PSMA, PSCA, and STEAP, and it is the first-in-human tested mRNA vaccine.^175^ Two clinical trials on the use of CV1903 in prostate cancer have been conducted. One (NCT00831467) investigated the effect of three increasing doses (256 mg, 640 mg, and 1280 mg total mRNA) in cohorts of three to six patients with prostate cancer, but the results are not published. Phase I of another open, phase I/II, uncontrolled, prospective study (NCT00906243) confirmed the safety of the dose of 320 µg RNA per antigen, providing a recommended dose for phase IIa to explore the immunological activity of that dose. Forty-four patients with increased PSA and mostly existing metastases (>80%) were recruited, and the results showed a superior immunogenicity rate induced by the vaccine in prostate cancer patients; antigen-specific T cells were observed in approximately 80% of patients independently of their HLA background, and approximately 58% reacted against multiple antigens. The PSA levels were stabilized in individual patients and dropped by more than 85% in one patient. One dose-limiting toxicity, urinary retention, was observed in six patients after the use of the highest dose. The most frequent adverse events were a reaction at the injection site or flu-like symptoms (e.g., chills and fever).

A subsequent clinical evaluation was performed due to the favorable safety profile and strong antigen-specific immune responses of CV9103 to assess two additional antigens, PAP and mucin-1, developing a new vaccine termed CV9104 that was used in two clinical studies.^175^ The first trial (NCT01817738) enrolled patients with castrate refractory metastatic prostate cancer who were subjected to surgery or androgen suppression therapy (by GNRH agonist or antagonist). The vaccination started at a dose of 1920 µg in weeks 1, 2, and 3, continued in weeks 5, 7, 9, 12, 15, 18, and 24, then every 6 weeks for 12 months and every 3 months thereafter until treatment discontinuation. The overall survival from the time of randomization was up to 3.5–4 years. The second trial using CV9104 (NCT02140138) was in an open-label randomized trial involving 35 high-risk and intermediate-risk patients with prostate cancer. Patients received four doses of CV9104 vaccine in weeks 1, 2, 3, and 5, and then, these patients underwent radical prostatectomy over one but within 2 weeks. The primary outcome was the evaluation of the antigen-specific cellular and humoral immune response to the vaccine, while the secondary outcome was the measurement of the incidence and severity of the adverse effects and the changes in PSA serum levels. However, no clinical therapeutic results have been provided thus far. Together, the mRNA vaccines against prostate cancer are mostly at an attempted stage, and the potential values in survival require more support from clinical results.

### mRNA vaccines against blood system cancer

Hematological malignancies encompass a range of diseases involving the abnormal proliferation of hematopoietic stem cells, including leukemia, myeloma, and lymphoma.^176^ Leukemia ranks as the first leading cause of cancer death among blood diseases and the tenth leading cause of cancer deaths overall worldwide (www.iarc.fr) according to the global cancer statistics for 2020 released by the International Agency for Research on Cancer. The common leukemia types include acute myeloid leukemia (AML), chronic myeloid leukemia, chronic myelomonocytic leukemia, chronic neutrophilic leukemia, and atypical chronic myeloid leukemia.^177^ mRNA vaccines have mainly been applied to AML in blood system cancer thus far. In 2005, Jarnjak-Jankovic showed that a DC-pulsed tumor mRNA vaccine triggered specific T-cell responses against leukemia cells in vitro.^178^ Subsequently, Driessche et al. reported a phase I clinical study with dose escalation of an autologous DC-pulsed mRNA vaccine encoding WT1 in 10 patients with AML.^179^ Patients were administered intradermal injections every 2 weeks, receiving 5, 10, or 20 × 10^6^ DC in the ventromedial region of the thigh or upper arm. The four doses were well tolerated by all patients, and no autoimmune or acute toxicities were observed throughout the entire trial. This team further showed the results of the use of the above vaccine in phase I/II study on AML patients.^180^ Patients with hematological remission after chemotherapy were enrolled 1 month after polychemotherapy for four biweekly vaccinations. Five (50%) patients (two of them refractory to chemotherapy) showed complete disease remission (absence of blasts in blood and less than 5% blasts in the bone marrow) after intradermal vaccination, with the myeloblast percentage decreasing to a normal level. Out of the five individuals, three exhibited long-term responses with complete remission that endured for over three years. A significantly positive correlation was found between the long-term response and WT1-specific CD8^+^ T-cell number. This study was performed again two years later on more patients, 17 in total, and among them, eight showed a complete response with a median relapse-free survival of 47 months.^181^ On this basis, this group reported a phase II trial about a DC-pulsed mRNA vaccine encoding WT1 as postremission treatment in 30 AML patients at high risk of relapse.^182^ Thirteen patients showed an antileukemic response, with five-year overall and relapse-free survival rates of 53.8% and 50%, respectively (7.7% and 30.8% in nonresponders, respectively). Patients aged ≤65 who had complete remission showed a longer 5-year survival (69.2%) than those aged > 65 years who experienced the same remission (30.8%), which is more than 51.7% in those aged ≤65 and 18% in those aged > 65 years present in the Swedish Acute Leukemia Registry. The same year, Khoury et al. also investigated a DC-pulsed mRNA vaccine encoding hTERT in 21 adult patients with AML: 16 in the first complete remission, three in the second complete remission, and two with early disease recurrence.^183^ Among those in complete remission, 11 (58%) developed a specific T-cell response and were free of disease at a median follow-up of 52 months. Four (57%) patients older than 60 years were free of disease recurrence at a median follow-up of 54 months. To improve the effectiveness exerted by mRNA vaccines targeting monoantigens, Lichtenegger et al. performed the first-in-human phase I trial involving 10 AML patients on TLR7/8-matured DC-pulsed mRNA vaccines encoding WT1 and PRAME (two AML-associated antigens) as well as CMV pp65.^184^ Seven patients were subjected to the complete regular 10 vaccinations, resulting in an increase in WT1 (2/10)-, PRAME (4/10)-, and CMV pp65 (9/10)-specific CD8^+^ T cells, and CMV pp65-induced CD4^+^ T cells (4/7) in the peripheral blood. The median relapse-free survival was 1084 days, while the median overall survival was not reached after 1057 days, with five patients (50%) relapse-free at the end of the observation.

Some studies have focused on chronic lymphocytic leukemia and lymphoma. The studies were mostly performed in preclinical trials. Kokhaei et al. showed that a DC-pulsed mRNA vaccine did not elicit a marked enhancement in IFN-γ-producing T cells compared with unpulsed DCs against B-cell chronic lymphocytic leukemia.^185^ In contrast, mRNA vaccines have exhibited effectiveness in lymphoma. In 2011, Fotin-Mleczek et al. reported the use of a two-component mRNA vaccine (protamine-complexed) encoding TLR7 and tumor antigen Gallus gallus OVA, HsPSMA, or HsSTEAP for treating T-cell lymphoma in an E. G7-OVA-based mouse model, in which E. G7-OVA is a mouse T-cell lymphoma cell line stably expressing Gallus gallus OVA.^186^ The vaccine triggered antigen-specific CD4^+^ and CD8^+^ T-cell responses, and sustained immune memory, and the vaccination mediated a strong antitumour response in both prophylactic and therapeutic contexts. In 2021, Tusup et al. conducted an assessment on the efficacy of an mRNA vaccine in inducing immune response against TCR CDR3 regions using a murine model based on EL4 T-lymphoma cell line, resulting in a feasible approach in protection against T-lymphoma.^187^ In 2022, Slam et al. developed an mRNA vaccine consisting of an OVA-coded mRNA and a palmitic acid-modified TLR7/8 agonist R848 (C16-R848) together with a lipid-polyethylene glycol shell.^174^ Vaccination significantly increased the amplification and infiltration of OVA-specific CD8^+^ T cells in OVA-expressing syngeneic allograft mouse models of lymphoma and prevented tumor growth when the vaccine was given before tumor engraftment (84% reduction vs. control). At present, a phase I study is ongoing for the evaluation of the effects of mRNA-2752 (a lipid nanoparticle encapsulating mRNAs encoding human OX40L, IL-23, and IL-36γ) alone and combined with an immune checkpoint blockade after intratumoural injection in solid tumors and lymphoma (NCT03739931).

Overall, the mRNA vaccine showed promising effects in AML in clinical trials, although none has been approved for its standard therapy. Except for AML, mRNA vaccines in other human blood system cancers are principally in the preclinical phase, and more clinical trials are warranted to investigate their efficacy.

### mRNA vaccines against digestive system cancer

Cancer can arise in any tissue of the gastrointestinal tract, including the colon, stomach, esophagus, liver, and pancreas.^188^ Digestive system cancer is a leading cause of cancer morbidity and death worldwide, and three million new cases and two million deaths from gastrointestinal cancers occur every year.^156^ Gholamin et al. used a DC-pulsed tumor mRNA vaccine in esophageal squamous cell carcinoma in vitro, and the results showed a significant induction of cytotoxicity (median >18.7% compared with the control) and INF-γ secretion (> twofold compared with the control).^189^ Mahdi Forghanifard et al. also used a DC-pulsed mRNA vaccine encoding MAGE-A4, NY-ESO1, and LAGE1, which also promoted the activation of CTLs against esophageal cells in vitro.^190^ Peng et al. used a DC-pulsed mRNA vaccine derived from HepG-2 cells or samples from hepatocellular carcinoma (HCC) patients, and the results showed an increase in the number of CD8^+^ T cells in cytotoxic T lymphocytes (CTLs) and a promotion of cytotoxic activity in HCC in vitro.^191^ A preclinical study using mRNA 5671 evaluated its therapeutic efficacy in colorectal cancer. The vaccine described encodes the four frequently observed KRAS mutations (G12C, G12D, G12V, and G13D).^192^ When used as monotherapy or in combination with pembrolizumab, it promotes an augmentation in CD8^+^ T-cell responses in mice. Similarly, Kim et al. showed that a DC-pulsed CEA mRNA vaccine with modification of calreticulin and the TAT protein transduction domain induced a potent CD4^+^ and CD8^+^ T-cell response and antitumour effects in mice with colon cancer.^193^ Clinically, Wan et al. used a CD40-B-cell-pulsed mRNA vaccine encoding alpha-fetoprotein for the treatment of HCC since their hypothesis was that the vaccine may boost a robust and prime naïve T-cell response;^194^ however, they did not report any preclinical or clinical results to date. Maeda reported a phase I clinical trial on a DC-pulsed heat-shock protein 70-encoded mRNA vaccine used at increasing doses in hepatitis C virus-related HCC.^195^ Twelve patients were enrolled, divided into three cohorts, and treated with three vaccinations every three weeks (1 × 10^7^, 2 × 10^7^, and 3 × 10^7^ DCs). The dose of 3 × 10^7^ DCs was the recommended dose according to the outcome of the pretreatment. Two patients experienced complete response without recurrence, five patients experienced disease progression, and five experienced stable disease. The two patients with complete response showed no disease recurrence for 44 and 33 months, respectively. Lesterhuis et al. conducted a comparison between the effects of DC-pulsed CEA peptide and DC-pulsed CEA mRNA vaccines in patients diagnosed with resectable liver metastases of colorectal cancer.^196^ All patients received three intravenous and intradermal vaccinations every week. However, anti-CEA-specific antibodies were detected in eight (8/11) patients in the peptide group, but no antibodies were found in the five patients in the mRNA group. In addition, an mRNA vaccine encoding neoantigens induced specific T-cell immune responses in patients with gastrointestinal cancer.^197^ The mRNA-based vaccine mRNA 4650 was clinically evaluated for the treatment of various digestive system cancers, including gastrointestinal cancer and liver cancer.^198,199^ Patients with gastrointestinal cancer treated with an intramuscular administration of mRNA 4650 developed CD4^+^ and CD8^+^ T-cell responses against tumor neoantigens. mRNA 4157 was designed to encode 34 unique neoantigens, and a phase I clinical study is ongoing in patients with MSI-high colorectal cancer and other solid tumors.^200^ It induces antigen-specific T cells and is well tolerated when used as monotherapy or in combination with pembrolizumab, leading to complete or partial responses. Suso et al. published a case report of a pancreatic cancer patient treated with a DC-pulsed telomerase-encoded mRNA vaccine.^201^ The patient was a 62-year-old woman who was treated with standard gemcitabine chemotherapy after developing multiple metastatic lymph node lesions after surgery. Chemotherapy was stopped because of the occurrence of severe neutropenia, and it was replaced with vaccination. The patient experienced a remarkable decrease in lymph node metastases after 32 months of vaccination without any increase in metabolic activity in the lesions compared with other lymph nodes. Furthermore, no serious treatment-related adverse events were observed during the 3-year vaccination. In 2013, Chen et al. compared the efficacy of DC-pulsed mRNA encoding mucin-4 and/or survivin in pancreatic cancer in vitro.^202^ All three cohorts induced a CTL response, which was stronger for DCs cotransfected with both antigens. A phase I clinical trial has been completed and evaluated the efficacy, safety, and tolerability in multiple cancers, including colorectal cancer, although the findings were not published (NCT03948763). In 2020, a phase I/II trial assessed the safety and immunogenicity of an mRNA-based, personalized vaccine against neoantigens in autologous gastrointestinal cancer (NCT03480152). Specific immunogenic mutations as targets for the mRNA vaccine were identified in tumor-infiltrating lymphocytes. The vaccination elicited a mutation-specific T-cell response against the predicted neoepitopes, but no objective clinical responses were found in the four treated patients in this trial. As mentioned in the paragraph on mRNA vaccines against NSCLC, a clinical study of personalized mRNA vaccines that encode tumor-specific antigens in patients with NSCLC and advanced esophageal cancer has been registered (NCT03908671), and the results in esophageal cancer are still unknown.

Altogether, although clinical trials using mRNA vaccines to combat digestive system cancer are limited, some effectiveness was shown in a fraction of patients, providing a foundation for further development of efficient treatments for digestive system cancer.

### mRNA vaccines against breast cancer

Breast cancer is the most frequently diagnosed cancer in women and the leading cause of cancer-related death globally.^156^ Global Cancer Statistics 2020 reports that female breast cancer has surpassed lung cancer and has become the most frequently diagnosed cancer.^156^ Breast cancer includes three major subtypes: ER^+^, HER2^+^, and triple-negative breast cancer (TNBC).^203^ Conventional endocrine or targeted drugs are not effective against TNBC compared with other subtypes, and TNBC has the worst prognosis, with over 50% of patients experiencing relapse within the initial 3 to 5 years following diagnosis and a median overall survival of 10.2 months.^203,204^ In 2013, five partners in academia and industry led by BioNTech AG launched The Mutanome Engineered RNA Immuno-Therapy project (NCT02316457) to validate a pioneering mRNA vaccine concept targeting individually expressed tumor antigens and tumor neo-antigens in patients with TNBC from clinical and industrial perspectives.^205,206^ This project developed a computational medicine platform to identify tumor neoantigens and TAAs in patients with TNBC, set up an mRNA vaccine warehouse for shared tumor antigens solving >95% of TNBC patients as well as a manufacturing process for producing a personalized mRNA vaccine. In addition, this platform evaluated the associated biomarkers identifying molecular and immunological signatures correlated with clinical events following vaccination and identified synergistic agents and optimized protocols of personalized vaccines. The vaccine consists of “off-the-shelf” mRNA selected from a presynthesized mRNA and a vaccine warehouse encoding neoantigens expressed in individual patient tumors as well as an mRNA engineered on-demand encoding patient-specific sequence stretches that incorporate nonsynonymous mutations. Every tumor is profiled before treatment to select the proper shared tumor antigens and detect mutations by exome sequencing. A cutting-edge platform is used for the design, manufacture, and release of tailored mRNA vaccines based on the output of the profiling. In 2019, Schmidt reported phase I/II trials assessing the feasibility, safety, and biological effectiveness of this personalized mRNA vaccine in Germany and Sweden.^206^ Patients were allocated to one of two study arms at the end of the standard of care therapy. Patients in arm 1 were subjected to eight vaccination cycles with a vaccine encoding shared TAAs selected according to the tumor antigen expression profile (mRNA WAREHOUSE vaccine). Patients in arm 2 were subjected to treatment with the mRNA WAREHOUSE vaccine followed by eight vaccination cycles with a vaccine encoding personalized 20 unique neoepitopes identified by next-generation sequencing (mRNA MUTATION vaccine). Preliminary immune response results from patients in arm2 have been disclosed at the Annual Meeting of the European Society of Medical Oncology.^206^ Vaccine-triggered CD4^+^ and/or CD8^+^ T-cell responses against 1-10 neoepitopes, as well as a great number of neoepitope-specific T-cell responses (10.3% of peripheral CD8^+^ T cells), were found in all 14 patients vaccinated with the mRNA MUTATION vaccine. Moreover, approximately 30% of peripheral CD8^+^ T cells exhibited a diversified CD8^+^ T-cell response, characterized by a high number of poly-epitopic TCR-clonotypes, which lasted for at least 6 months at high levels after the last vaccination. Although vaccination induced specific T-cell responses, the survival data are still unpublished, and the efficacy of the mRNA vaccine is not yet clear. Moreover, only one study investigated the efficacy of personalized anti-breast cancer mRNA vaccines, and more trials are needed to promote them in clinical practice.

## mRNA vaccines in immunological diseases

Autoimmune diseases are characterized by chronic inflammation due to a dysregulated immune response to self-antigens.^207^ Many clinical studies using mRNA vaccines against cancers or infectious diseases have exhibited their potential to trigger autoimmune diseases.^8^ However, mouse models have revealed their ability to treat autoimmune diseases, although no clinical applications have yet been performed.^208^ The physiological induction and maintenance of peripheral tolerance are primarily determined by the presentation of self-antigens by antigen-presenting cells (APCs) with diminished surface expression of costimulatory molecules, such as DC86. Conventional U-composed mRNA vaccines often elicit strong type I T helper cell responses driven by TLR signaling. Krienke et al. introduced a liposomal formulation that systemically delivers antigens encoded by the mRNA vaccine into lymphoid tissue-resident CD11c^+^ APCs and replaced uridine (U) by the incorporation of N1-methylpseudouridine. This method avoids the significant activation of CD8^+^ T cells, CD4^+^ T cells, CD11^+^ APCs, natural killer cells, and B cells, as well as the secretion of IFN-α or other inflammatory cytokines in mice, suggesting that nanoparticle-formulated N1-methylpseudouridin-modified mRNA is appropriate for the noninflammatory delivery of proteins into splenic CD11c^+^ APCs. In an experimental autoimmune encephalomyelitis mouse model of multiple sclerosis induced by the selective expression of MOG (the epitope of myelin oligodendrocyte glycoprotein in DCs), mice were vaccinated with MOG-encoding N1-methylpseudouridine mRNA after immunization with MOG, and the results showed that they were protected from disease development. Vaccination also prevented further disease progression in mice with an established disease and even reverted pathology in some cases. The treatment suppressed disease-promoting TH1, TH17, and TH1/TH17 cells by inducing FOXP3^+^ regulatory T cells and increasing the expression of T-cell exhaustion markers (e.g., PD-1, CTLA4, TIGIT, TIM-3, and LGA-3). Vaccination did not influence the immune responses to unrelated antigens, and this approach was effective in models induced by different antigens (PLP, MBP, and MOBP), suggesting important aspects of this approach, such as the possibility of optimizing the mRNA vaccine to elicit protective immune responses against specific pathologies and maintaining antigen-specific immune tolerance to treat autoimmune diseases.

Allergy is a hypersensitivity reaction of the immune system to a foreign substance that is typically harmless to most individuals. This foreign substance, known as an allergen, triggers an immune response that results in various symptoms, such as itching, sneezing, watery eyes, and skin rash. Common allergens include pollen, dust mites, certain foods, medications, and insect venom. Allergies can range from mild to severe and, in some cases, can be life-threatening. mRNA vaccines also offer a safer approach to preventing allergic conditions by encoding the allergen and providing a purer immunizing antigen compared to traditional allergen extracts.^209^ In mice, mRNA vaccines that encode allergens have been found to be effective in preventing type I allergies by activating a Th1 cell response.^210^ After immunization, the mice were exposed to the corresponding allergen, and the resulting inflammatory signatures (e.g., eosinophils, IL-4 and IL-5) were reduced, while anti-inflammatory responses were enhanced (e.g., the induction of IFN-γ-producing cells).^211^ More importantly, mRNA vaccines have been exhibited to generate long-term memory responses in mice, leading to potent anti-inflammatory responses upon re-exposure to allergens.^212^ These findings illustrate the potential of mRNA vaccines for targeting allergies without the need for booster vaccinations.

## mRNA vaccines in tissue damage

Tissue damage refers to any physical injury or harm that occurs to the body’s tissues. This can be resulted from a variety of factors, such as trauma, infection, inflammation, and exposure to harmful substances or radiation. Tissue damage can affect any part of the body, including the skin, muscles, bones, organs, and nerves. Cardiovascular damage is the leading threat to human health worldwide.^173^ They include but are not limited to coronary heart disease, hypertension, heart failure, vascular calcification, and cardiac fibrosis.^139^ Cardiovascular damage is mostly irreversible and can only be controlled. In 2016, AstraZeneca developed AZD8601, an mRNA vaccine encoding VEGF-A165 with a minimal innate immune response.^213–215^ AZD8601 used in preclinical models induced more blood vessels in local tissue and significantly accelerated the healing of chronic wounds in a dose-dependent manner.^213–215^ A clinical trial was subsequently started in 2017 in patients with coronary artery disease undergoing coronary artery bypass grafting surgery (NCT03370887). Patients were randomly and equally divided into three groups and further treated with AZD8601 at different doses or placebo, with the evaluation of the safety of AZD8601 as the primary endpoint. The results were reported at the American Heart Association’s Scientific Sessions 2021, showing the safety and tolerability of AZD8601 as well as the positive trends in exploratory efficacy objectives. Rurik et al. developed an antifibrotic treatment strategy based on chimeric antigen receptor T cells using CD5-targeted LNPs-mRNA. Ten micrograms of CD5/LNP-mRNA encoding FAPCAR were intravenously injected into mice with cardiac injury induced by the delivery of AngII/PE. Echocardiography showed remarkable functional improvement in the injured mice 2 weeks after the initial treatment. Of note, left ventricular diastolic function was significantly improved and returned to the original healthy level during the follow-up period. The improvement in the extracellular matrix burden was more evident in the mice treated with LNP-mRNA than in those treated with saline. Altogether, these findings were encouraging and provide possibilities for the treatment of irreversible cardiovascular diseases.

Apart from cardiovascular diseases, mRNA vaccines have shown effectiveness in multiple soft tissue damages.^216^ The administration of mRNA-LNPs containing nucleoside-modified mRNA that encodes HGF and EGF was found to stimulate liver regeneration in mice with chronic choline-deficient ethionine-mediated liver injury and acute acetaminophen-induced liver toxicity.^217^ In the same year, another study utilized mRNA that encodes VEGF-C to induce the growth of lymphatic vessels in mice.^218^ By administering low dose of VEGF-C mRNA-loaded lipid nanoparticles (mRNA-LNPs), targeted lymphatic growth was induced, leading to the remarkable reversal of lymphedema and restoration of lymphatic function in an experimental mouse model. In a mouse model of diabetes, the delivery of nucleoside-modified mRNA encoding FGF-2 through mineral-coated microparticles improved the healing of dermal wounds by hastening the process of complete wound closure.^219^

In 2015, Elangovan et al. showcased the promising potential of mRNA-based therapeutic strategies in the field of bone regeneration.^220^ They employed pseudouridine and 5-methylcytidine-modified mRNA encoding BMP-2, which was combined with polyethylenimine (PEI) and incorporated into collagen scaffolds prior to the implantation into rat calvarial defects. After a duration of 4 weeks, the PEI-BMP-2 mRNA-activated matrices exhibited a significant improvement in bone regeneration when compared with the PEI-complexed BMP-2 pDNA-activated matrices. Microcomputed tomography analysis revealed a significant increase in both the amount of bone volume and total volume of regenerated bone in defects treated with scaffolds embedded with PEI-mRNA and PEI-pDNA complexes. Specifically, the defects treated with PEI-mRNA exhibited a 3.9-fold higher bone volume, while the total volume of regenerated bone was 1.9-fold higher compared to the negative control group. Balmayor et al. also confirmed the osteogenic potential of nucleoside-modified BMP-2 mRNA treatment in a rat femur bone defect model.^221^ Furthermore, the administration of a low dose (2.5 µg/defect) of nucleoside-modified mRNA within a fibrin gel matrix demonstrated speeded up bone healing compared to the fibrin control group, as evidenced by significant improvements observed just 2 weeks after application. A study was undertaken with the goal of augmenting long-lasting mRNA delivery to specific cells and creating a convenient ready-to-use product. To achieve this, the researchers developed a vacuum-dried construct known as transcript-activated matrices (TAMs). In a noncritical femoral bone defect rat model, collagen sponges were preloaded with nucleoside-modified BMP-2 mRNA-loaded lipid nanoparticles (mRNA-LNPs), resulting in a remarkable enhancement of bone generation when compared to empty collagen sponges. exhibited exceptional stability at room temperature for a minimum of 6 months, and facilitated prolonged protein generation for up to 6 days. This seminal study showed BMP-2-encoding TAMs were effective in delivering sustained mRNA to target cells. In a subsequent investigation, the researchers explored the dose-dependent impact of nucleoside-modified BMP-2-encoding TAMs on the promotion of new bone formation in a critical femoral defect rat model. Micro-CT and histological analyses revealed that the higher dose of the product (15 µg/defect) exhibited approximately double the amount of newly formed bone compared to the lower dose (3.75 µg/defect).^222^ A study conducted a comparison of BMP-9-PEI-activated matrix (collagen scaffold) and BMP-2-PEI-activated matrix in terms of their ability to promote bone regeneration. The results unveiled a superior capacity of BMP-9 mRNA transfection in enhancing the in vitro osteogenic differentiation of human bone marrow mesenchymal stem cells compared to the administration of BMP-2 mRNA. Furthermore, when implanted in rat calvarial bone defects, BMP-9 mRNA exhibited a remarkable 2-fold increase in the connectivity density of the regenerated bone compared to BMP-2 mRNA.^223^ To enhance the gene-activated collagen membrane, an additional improvement was made by immersing the perforated collagen membrane in a solution containing BMP-9 mRNA-PEI complexes, followed by a freeze-drying process. Upon application of this product to rat calvarial defects, a notable and significant formation of new bone was observed after a 4-week period of treatment.^224^ A combination therapy involving mRNA, stem cell transplantation, and scaffolds has recently been investigated for bone regeneration. In a rat model of calvarial bone defects, the implantation of nucleoside-modified BMP-2 and VEGF-A mRNA-transfected bone marrow mesenchymal stem cells within a collagen scaffold resulted in a significant augmentation of bone regeneration. The simultaneous delivery of BMP-2 and VEGF-A mRNAs exhibited a synergistic effect, effectively promoting both osteogenic and angiogenic processes.^225^ This synergistic action resulted in superior healing outcomes when compared to treatments involving BMP-2 or VEGF-A alone. The findings strongly indicate that employing a combination of multiple growth factor-encoding mRNAs, along with cell therapy and a biomaterial scaffold, holds great promise as a viable strategy to attain favorable outcomes for bone regeneration.

Together, mRNA vaccines show promising potential in the promotion of tissue generation. Apart from the abovementioned damage, mRNA vaccines may be able to promote the generation of other tissues.

## mRNA vaccines in rare diseases

Rare diseases are defined as medical conditions that impact a small proportion of the population, characterized by their low prevalence and often limited understanding due to their rarity. Patients may struggle to find appropriate medical care and treatment. mRNA vaccines have been reported to have the potential to treat multiple rare diseases. Cystic fibrosis is a hereditary condition, predominantly impacting the lungs, pancreas, and other organs.^226,227^ It is caused by a mutation in the *CFTR* gene, causing the generation of thick, sticky mucus in the lungs and other organs. This mucus can clog airways and make it difficult to breathe, leading to chronic lung infections, lung damage, and respiratory failure. Cystic fibrosis can also affect the pancreas, causing digestive problems and malnutrition, and it can lead to other complications, such as liver disease, diabetes, and infertility. Cystic fibrosis is a lifelong condition that currently has no cure, while treatment helps symptom management as well as improves quality of life. In 2018, Robinson et al. reported that a clinically relevant lipid nanoparticle-packed chemically modified mRNA encoding CFTR increased membrane-localized CFTR and rescued its role as a chloride channel in patient-derived bronchial epithelial cells; its nasal application restored CFTR-mediated chloride secretion to conductive airway epithelia in *CFTR*-deficient mice, representing a promising platform for the correction of cystic fibrosis.^228^ Preclinical evaluation of MRT5005 (an mRNA encoding the CFTR protein) administered by nebulization validated cystic fibrosis correction in mice and nonhuman primates.^229^ A phase I/II clinical study is currently in progress, seeking participants for a randomized, double-blinded, placebo-controlled study. The trial aims to assess the safety, tolerability, and biological activity of MRT5005 when administered via nebulization to adults diagnosed with cystic fibrosis (NCT03375047).

Inherited metabolic disorders are significant contributors to illness and death in children.^230^ These disorders, which affect approximately 1 in 800 live births, often stem from mutations in a single gene inherited in an autosomal recessive pattern.^231^ Inherited metabolic diseases are responsible for 10–15% of pediatric acute liver failure cases, with mortality rates ranging from 22–65%.^231^ mRNA vaccines have been tested in several rare genetic disorders, such as hereditary tyrosinemia type 1, phenylketonuria (PKU), methylmalonic acidemia (MMA), propionic acidemia (PA), glycogen storage disease type 1a (GSD1a), and ornithine transcarbamylase (OTC) deficiency. PKU is a genetic metabolic disorder resulting from insufficient functional phenylalanine hydroxylase (PAH) activity, causing the buildup of phenylalanine (Phe) in the blood and organs of those affected.^232,233^ Without treatment, patients experience significant neurological damage. Administering mouse Pah mRNA packaged in LNPs through repeated intravenous injection into a PKU (Pah^enu2^) mouse model produced therapeutic PAH protein, reduced Phe levels in the liver, serum, and brain, and reversed the progression of the disease.^234,235^ These findings suggest Pah mRNA formulated in LNPs offers an alternative therapeutic option for PKU patients who eliminates the need for a lifelong Phe-restricted diet. In line with this possibility, ModernaTx, Inc. (Cambridge, MA, USA) has included PAH PKU mRNA-3283 in its product development pipeline (www.modernatx.com/research/product-pipeline). MMA is an organic acidaemia that poses a high risk of morbidity as well as death and currently has no approved treatments addressing its underlying cause.^236^ This autosomal recessive disorder hinders the metabolism of propionate derived from certain proteins and fats.^237^ As a result, there is a notable accumulation of methylmalonic acid in body fluids and tissues. The primary cause of this disease is commonly attributed to a deficiency in the mitochondrial enzyme known as methylmalonyl-coenzyme A (CoA) mutase (MUT). Repeated intravenous injection of LNP-encapsulated MUT mRNA into hypomorphic Mut^−/−^, Tg^INS-CBA-G715V^ mice resulted in a decrease in plasma MMA concentrations as well as an enhanced survival rate.^238,239^ Significantly, comprehensive safety studies revealed no discernible alterations in liver function tests, inflammatory cytokine generation, or the production of anti-MMA antibodies. A phase I/II clinical trial is presently underway to assess the safety, pharmacokinetics, and pharmacodynamics of administering LNP-encapsulated human MUT mRNA (mRNA-3705) to individuals diagnosed with isolated methylmalonic acidemia (NCT04899310 and NCT05295433). PA is a pediatric disorder caused by a mitochondrial deficiency in propionyl-CoA carboxylase (PCC), which is an enzyme consisting of a heterododecamer encoded by the *PCCA* and *PCCB* genes that plays a vital role in catalyzing the carboxylation of propionyl-CoA to methylmalonyl-CoA within the body.^240^ This deficiency hampers the metabolism of propionate, resulting in the accumulation of toxic metabolites within the body, such as 2-methylcitrate, 3-hydroxypropionate, and propionyl carnitine. Intravenous injection of LNP-encapsulated PCCA and PCCB mRNAs led to the generation of therapeutic levels of PCCA and PCCB in the livers of a hypomorphic disease model (Pcca^−/−^[p. A138T]) in mice.^241^ During a 6-month duration, the repeated administration of PCCA and PCCB mRNAs encapsulated in LNPs was well tolerated. This treatment approach resulted in a reduction of toxic metabolite levels in the plasma, although complete normalization was not achieved. Liver transaminase levels remained within the normal range, and no adverse reactions were observed. These findings support the ongoing Phase I/II study of mRNA-3927 (LNP-encapsulated PCCA and PCCB mRNAs) to evaluate the safety and pharmacodynamic activity of the therapy in PA patients aged 1 year or older (NCT05130437 and NCT04159103). GSD1a is a genetic metabolic disorder resulting from an autosomal recessive mutation in the gene responsible for coding the catalytic subunit of glucose-6-phosphatase (G6Pase).^242^ This enzyme hydrolyses glucose-6-phosphate, producing free glucose. As the main hub for gluconeogenesis, the liver serves as the primary organ affected by disruptions in this process. GSD1a is characterized by symptoms such as hypoglycemia, hypertriglyceridaemia, anemia, renal disease, and an increased lifelong risk of HCC. A recent study demonstrated that repeated intravenous injection of LNP-encapsulated hG6PC-a mRNA in a liver-specific G6pc knockout mouse (L. G6pc^−/−^) resulted in a significant enhancement in fasting glycemia and a decrease in GSD1a biomarkers, such as glycogen, G6P, and triglycerides.^243^ Both treated and control animals exhibited similar levels of cytokines, including IFN-γ, IL-1β, TNFα, and IL-6, in their serum. The treatment did not induce anti-G6Pase responses, liver injury, alterations in body weight, or any signs of distress. These results support further investigation of LNP-encapsulated mRNA as a potential treatment for inherited metabolic disorders. Currently, a clinical study is underway to assess the safety, tolerability, pharmacokinetics, and pharmacodynamics of a single intravenous dose of LNP-encapsulated hG6PC-a mRNA (mRNA-3745) in patients with GSD1a (NCT05095727). OTC is a crucial enzyme in the urea cycle that is found in the liver and facilitates the conversion of carbamoyl phosphate and ornithine into citrulline and phosphate.^244^ This process plays vital roles in the elimination of ammonia from the body. High levels of ammonia can cause varying degrees of neuropsychiatric symptoms. Despite various available treatments, such as protein-restricted diets and ammonia scavengers, it is important to note that there is currently no definitive treatment for addressing the root cause of OTC deficiency. Prieve et al. demonstrated that NP-encapsulated hOTC mRNA (ARCT-810) successfully treated a hyperammonemic murine model of OTC deficiency (Otcspf-ash), resulting in the normalization of plasma ammonia and orotic acid levels, an enhanced survival, as well as a good safety profile.^245^ A phase I study has been completed assessing the safety, tolerability and pharmacokinetics of ARCT-810 in healthy adult subjects, but the result has not been reported (NCT04416126). Two phase IB clinical trials (NCT05526066 and NCT04442347) are currently underway to assess the safety, tolerability, and pharmacokinetics of a single dose of ARCT-810 in clinically stable OTC-deficient patients.

Together, there is a lack of therapeutic agents that can cure these rare diseases. mRNA vaccines render it possible to control these diseases long-term, despite still in an attempt stage. More studies are warranted to validate their efficacy against rare diseases.

## Conclusions and perspectives

mRNA vaccines have become a hotspot in disease prevention and treatment, becoming predominant in preclinical and clinical trials, especially in infectious diseases and cancers.^141^ Nevertheless, except for the anti-COVID-19 mRNA vaccine, few have been approved for disease treatment thus far. Several challenges are not completely addressed that may limit the application of mRNA vaccines. Striking a balance between achieving optimal antigen production and ensuring adequate adjuvant effects poses a significant challenge. The adjuvant effect of mRNA vaccines promotes innate and adaptive immunity, but excessive innate immunity inhibits mRNA translation.^8,246^ 5′ capping, nucleoside modification, poly(A) tail modification, and HPLC purification are strategies already used to decrease innate immunity.^17,247,248^ The interaction of the delivery carrier mRNA and innate immune system requires further investigation to achieve an effective balance. Another challenge is the large-scale manufacturing of mRNA. As a consequence of the lack of a continuous manufacturing process, synthesis, purification, and formulation must be performed in different facilities in three states in the USA, largely limiting the rapid manufacture of mRNA vaccines. For instance, the manufacture of millions of doses of BNT162b2 takes 60 days, far from satisfying the vaccination needs of 6 billion people worldwide (derived from https://www.nytimes.com/interactive/2021/health/pfizer-coronavirus-vaccine.html). A continuous manufacturing process may enhance the efficiency of mRNA vaccine production by combining three facilities into a fluidic system. Continuous manufacturing may ensure the recycling and reuse of raw compounds (e.g., enzymes or NTPs), and avoiding transport may significantly reduce time and costs. Proper temperature control is crucial for maintaining the efficacy of vaccines. Most vaccines can be stored at 2–8 °C for extended periods, and mRNA vaccines such as BNT162b2 and mRNA-1273 must be kept at −80 °C and −20 °C, respectively. This poses a significant challenge for their distribution. The instability of the LNP-mRNA system is the reason for the strict temperature requirement for storing mRNA vaccines. Despite various lyoprotectants (e.g., lactate, mannose, and trehalose) have been incorporated into mRNA-protamine formulations, enabling successful long-term storage at room temperature after freeze-drying, as claimed in several patents, it is important to note that the efficacy of preserving mRNA delivery efficiency in vivo has been limited when 20% (weight by volume) sucrose or trehalose is added to LNPs and subjected to freeze-drying. The alteration of the nanostructure of the LNP-mRNA system due to freeze-drying and reconstitution is believed to potentially impact the LNPs’ interactions with plasma, which can lead to a decline in mRNA delivery efficiency in vivo. To date, there is no known resolution to the requirement for extremely cold storage and transportation conditions for LNP-mRNA vaccines, which could impose significant constraints on the widespread use of mRNA vaccines in the future. Safety is another concern in the use of mRNA vaccines. The extensive deployment of the COVID-19 mRNA vaccine created a chance to thoroughly study the adverse reactions associated with mRNA vaccines. According to safety monitoring by the Centers for Disease Control and Prevention (CDC, https://www.cdc.gov/coronavirus/2019-ncov/vaccines/safety/adverse-events.html), some people have reported no side effects after administration of the COVID-19 mRNA vaccine, while many have experienced mild to moderate side effects such as headache, fatigue, and soreness at the injection site, which are generally temporary and typically resolve within a few days. Although several reactions are rare after vaccination, multiple cases have been reported. Anaphylaxis, a severe type of allergic reaction, has occurred in about 5 cases per million vaccine doses administered. Thrombosis with thrombocytopenia syndrome is a rare yet significant adverse event characterized by the formation of blood clots in major blood vessels and a decrease in platelet count. It has been reported in around 4 cases per million doses administered, signifying its infrequent occurrence but considerable severity. More importantly, the cases of myocarditis and pericarditis are increasing after the administration of mRNA vaccines. During the study period, more than 350 million mRNA vaccines were administered, and the CDC scientists observed that the incidence of myocarditis was highest among males in the following age groups following the second dose of an mRNA vaccine: 12–15 years (70.7 cases per million doses of Pfizer-BioNTech), 16–17 years (105.9 cases per million doses of Pfizer-BioNTech), and 18–24 years (52.4 cases and 56.3 cases per million doses of Pfizer-BioNTech and Moderna, respectively). As of March 2, 2023, 715 reports have been verified to meet the CDC’s working case definition for myocarditis, and the findings are as follows: 5–11 years (23 verified reports of myocarditis after 23,376,785 doses administered), 12–15 years (376 verified reports of myocarditis after 25,913,772 doses administered), and 16–17 years (316 verified reports of myocarditis after 14,180,263 doses administered). The mechanisms causing these rare adverse events remain to be addressed. Finally, the durability of mRNA vaccines against COVID-19, such as the Pfizer-BioNTech and Moderna vaccines, may decline over time. The virus is constantly evolving, and new variants may emerge that are not as well recognized by the immune system as the original virus, leading to decreased effectiveness of the vaccine over time, especially if the variants become more prevalent. In addition, the immune response generated by the vaccine may decrease over time as the immune system’s memory of the virus fades. This is a normal process that occurs with any vaccine, but the rate of decline may be faster with mRNA vaccines due to their unique mechanism of action. Furthermore, the vaccine may not provide as strong or long-lasting protection against certain populations, such as immunocompromised individuals or elderly individuals. Several approaches may improve the overall effectiveness of the vaccine and extend its duration, including administering booster shots of the mRNA vaccine at specific intervals, using different types of vaccines (such as a combination of mRNA and traditional vaccines), and optimizing the storage and transportation conditions for mRNA vaccines. Altogether, the technique for mRNA vaccine preparation and application is not perfect and remains to be further ameliorated.

In addition to these universal issues underlying mRNA vaccines, there are specific challenges in different diseases. Given the application of mRNA vaccines in immunological diseases, rare diseases, and tissue damage are still at an early stage, and there are insufficient studies assessing their efficacies and challenges in the context of these diseases. Therefore, infectious diseases and cancer, in which mRNA vaccines are more prevalently used, are selected as examples for discussing the obstacles of mRNA vaccines in specific diseases. There are two main categories of infectious viruses: those that are newly emerging or reemerging and those that cause chronic infections. The protection efficacy of mRNA vaccines against the rapidly emerged coronavirus has been exceptional, and their low production cost and ease of manufacture suggest that they could be instrumental in control of future pandemics resulted from rapidly emerging viruses. However, these emerging or reemerging viruses tend to mutate rapidly, presenting a challenge in developing mRNA vaccines that are broad or seasonal in nature. Additionally, generating effective neutralizing antibodies against chronic infectious viruses is typically difficult, as they are adept at evading innate immunity. Unlike infectious diseases, cancer is caused by genetic and epigenetic factors, and it is characterized by complex and heterogeneous antigen expression, thus requiring the use of a personalized mRNA vaccine. However, several challenges limit the clinical application of personalized cancer mRNA vaccines, such as the still technological obstacles limiting the precise detection and quantification of immunogenic tumor neoantigens and an insufficient understanding of the accurate biological mechanism of tumor immune evasion. Conventional exome sequencing does not capture noncanonical peptides derived from the genomic “dark matter” that may include most of the new epitopes expressed by tumors.^249,250^ Experimental and in silico approaches for identifying neoantigens are largely biased toward MHC I epitopes and insensitive to MHC II and rare allotypes, causing a significant underestimation of the frequency of targetable immunogenic neoantigens. Moreover, a therapeutic vaccine usually works better in the context of adjuvant therapy or in cases of minimal residual disease, where the tumor burden is low and the immunosuppressive microenvironment is not firmly established.^251^ Instead, the T-cell response triggered by personalized vaccination would be largely slowed down by various immunosuppressive cells^252–254^ (e.g., cancer-associated fibroblasts, vascular endothelial cells, tumor-associated macrophages, tumor-associated neutrophils, suppressive myeloid cells, regulatory T cells, and regulatory B cells) and immunosuppressive regulators (e.g., PD-1, PD-L1, CTLA-4, IDO-1, TGF-β, IL-10, and IL-35) in the tumor immune microenvironment (TIME) of large load tumors. In this context, a combined therapy is required for effective control of tumors. Vaccination enables the turn from the immunological “cold” tumor into the “hot” phenotype and induces PD-L1 upregulation in the TIME.^251^ This phenomenon guides the combination of PD-1/PD-L1 blockade and personalized vaccination. The clinical trial NCT03897881 evaluating pembrolizumab in combination with neoantigen vaccination against melanoma is active but not recruiting patients; for example, the study is ongoing, and the participants are under therapy or being evaluated but not enrolled. Similarly, cancer vaccines preclinically synergize with the inhibition of other inhibitory molecules (e.g., CTLA-4, TIM-3, LAG-3, IDO, or TGF-β) and the stimulation of costimulatory molecules (e.g., GITR, OX40, and CD137).^251^ Additionally, a phase I clinical trial for glioblastoma (NCT02709616) tested a personalized vaccination together with temozolomide and radiotherapy. Recently, Huang et al. established a pipeline to construct tumor immune subtypes, which act as biomarkers that reflect the immune status in tumors and their TIME (e.g., immune infiltration and function, as well as the expression of immune checkpoints and immunological cell death modulators).^255,256^ The immune subtype might provide precise guidance for combined cooperation with the mRNA vaccine, warranting further clinical investigation.

### Supplementary information

MS-R2-Similarity check

Authorship+form-R2
