# Supplementary material for: mRNA vaccines in disease prevention and treatment
Source: Signal Transduct Target Ther. 2023 Sep 20;8:365. doi: 10.1038/s41392-023-01579-1 (PMC10509165; doi:10.1038/s41392-023-01579-1)
Supplement: Supplementary file 2 — Authorship+form-R2 [file 41392_2023_1579_MOESM2_ESM.pdf]

SPRINGER NATURE

Change of authorship request form - Journals (pre-acceptance)

Important information. Please read.

- This form should be used by authors to request any change in authorship (adding/deleting authors) including changes in corresponding authors. This form should not be used for name changes. Please fully complete all sections. Use black ink and block capitals and provide each author's full name with the given name first followed by the family name.

- By signing this declaration, all authors guarantee that the order of the authors are in accordance with their scientific contribution, if applicable as different conventions apply per discipline, and that only authors have been added who made a meaningful contribution to the work.

- Please note, in author collaborations where there is formal agreement for representing the collaboration, it is sufficient for the representative or legal guarantor (usually the corresponding author) to complete and sign the Authorship Change Form on behalf of all authors, **next to the added/removed author(s)**. **(Complete Section 3, followed by Section 6.)**
  In author collaborations where there is no formal agreement for representing the collaboration and **there are more than 10 authors**, one may sign for all, provided the signer appends correspondence that attests that each of the authors have agreed to the change **and the added/removed authors sign the form**. **(Complete Section 3, followed by Section 6.)**

- Please note, we cannot investigate or mediate any authorship disputes. If you are unable to obtain agreement from all authors (including those who you wish to be removed) you must refer the matter to your institution(s) for investigation. Please inform us if you need to do this.

- If you are not able to return a fully completed form within **30 days** of the date that it was sent to the author requesting the change, we may have to withdraw your manuscript. We cannot publish manuscripts where authorship has not been agreed by all authors (including those who have been removed).

- Incomplete forms will be rejected.

- Please return/upload this form, fully completed, to the Journals Editorial Office. The Journal and/or Publisher will consider the information you have provided to decide whether to approve the proposed change in authorship. We may decide to contact your institution for more information or undertake a further investigation, if appropriate, before making a final decision.

SPRINGER NATURE

Change of authorship request form - Journals (pre-acceptance)

Section 1: Please provide the current title of manuscript

Manuscript ID no.: SIGTRANS-08927

Title: mRNA vaccine in disease prevention and treatment

Section 2: Please provide the previous authorship, in the order shown on the manuscript before the changes were introduced. Please indicate the corresponding author by adding (CA) behind the name.

	First name(s)	Family name	ORCID or SCOPUS id, if available
1st author	Xing	Huang (CA)	http://orcid.org/0000-0002-8886-2777
2nd author	Gang	Zhang	
3rd author	Tian-yu	Tang	
4th author	Ting-bo	Liang (CA)	
5th author			
6th author			
7th author			
8th author			
9th author			
10th author			

Please use an additional sheet if there are more than 10 authors.

SPRINGER NATURE Springer Nature is one of the world's leading global research, educational and professional publishers, created in May 2015 through the combination of Nature Publishing Group, Palgrave Macmillan, Macmillan Education and Springer Science+Business Media.

Change of authorship request form - Journals (pre-acceptance)

SPRINGER NATURE

Section 3: Please provide a justification for change. Please use this section to explain your reasons for changing the authorship of your manuscript, e.g. what necessitated the change in authorship? Please refer to the (journal) policy pages for more information about authorship. Please explain why omitted authors were not originally included and/or why authors were removed on the submitted manuscript.

Given Gang Zhang has made a great contribution on literature review and writing as well as rephrasing and proof-reading, he has been listed as the first author in the manuscript.

Given Yin-feng Chen has made a great contribution on rephrasing and proof-reading, he has been listed as a co-author in the manuscript.

Section 4: Proposed new authorship. Please provide your new authorship list in the order you would like it to appear on the manuscript. Please indicate the corresponding author by adding (CA) behind the name. If the Corresponding Author has changed, please indicate the reason under section 3.

	First name(s)	Family name (this name will appear in full on the final publication and will be searchable in various abstract and indexing databases)	Affiliated institute	E-mail address
1st author	Gang	Zhang	Zhejiang University School of Medicine	Gang_Zhang@zju.edu.cn
2nd author	Tian-yu	Tang	Zhejiang University School of Medicine	tangtianyu0701@zju.edu.cn
3rd author	Yin-feng	Chen	Zhejiang University School of Medicine	chenyinfeng651@163.com
4th author	Xing	Huang (CA)	Zhejiang University School of Medicine	huangxing66@zju.edu.cn
5th author	Ting-bo	Liang (CA)	Zhejiang University School of Medicine	liangtingbo@zju.edu.cn
6th author				
7th author				
8th author				
9th author				
10th author				

Please use an additional sheet if there are more than 10 authors.

SPRINGER NATURE Springer Nature is one of the world's leading global research, educational and professional publishers, created in May 2015 through the combination of Nature Publishing Group, Palgrave Macmillan, Macmillan Education and Springer Science+Business Media.

SPRINGER NATURE

Change of authorship request form - Journals (pre-acceptance)

Section 5: Author contribution, Acknowledgement and Disclosures. Please use this section to provide a new disclosure statement and, if appropriate, acknowledge any contributors who have been removed as authors and ensure you state what contribution any new authors made (if applicable per the journal or book (series) policy). Please ensure these are updated in your manuscript - after approval of the change(s) - as our production department will not transfer the information in this form to your manuscript.

New acknowledgements:

This work was supported by the National Natural Science Foundation of China (31970696 and U20A20378), Zhejiang Provincial Natural Science Foundation for Distinguished Young Scholar (LR22H160010), National Key Research and Development Program (2019YFC1316000), Zhejiang Provincial Key Research and Development Program (2019C03019), and Zhejiang Provincial College Student Science and Technology Innovation Activity Plan-College Student Innovation and Entrepreneurship Incubation Program (2023R401203 to G.Z.).

New Disclosures (financial and non-financial interests, funding):

The authors declare that they have no competing interests.

New Author Contributions statement (if applicable per the journal policy):

X.H. and T.L. conceived the manuscript and share senior authorship. X.H., G.Z., and T.T. contributed equally to the literature review and writing. G.Z. and Y.C. contributed to rephrasing and proof-reading. All authors discussed and edited the manuscript and approved the final version.

State 'Not applicable' if there are no new authors.

SPRINGER NATURE Springer Nature is one of the world's leading global research, educational and professional publishers, created in May 2015 through the combination of Nature Publishing Group, Palgrave Macmillan, Macmillan Education and Springer Science+Business Media.

SPRINGER NATURE

Change of authorship request form - Journals (pre-acceptance)

Section 6: Declaration of agreement. *All* authors, unchanged, new and removed *must* sign this declaration.
(NB: Please print the form, (docu)-sign and return/upload a scanned copy. Please note that signatures that have been inserted as an image file are acceptable as long as it is handwritten. Typed names in the signature box are unacceptable.) *** Please delete as appropriate. Delete all of the bold if you were on the original authorship list and are remaining as an author.**

	First name	Family name		Signature	Date
1st author	Gang	Zhang	I agree to the proposed new authorship shown in section 4	Gang Zhang	July 1st, 2023
2nd author	Tian-yu	Tang	I agree to the proposed new authorship shown in section 4	Tianyu Tang	July 1st, 2023
3rd author	Yin-feng	Chen	I agree to the proposed new authorship shown in section 4	Yinfeng Chen	July 1st, 2023
4th authors	Xing	Huang	I agree to the proposed new authorship shown in section 4	Xing Huang	July 1st, 2023
5th author	Ting-bo	Liang	I agree to the proposed new authorship shown in section 4	梁廷波	July 1st, 2023
6th author					
7th author					

SPRINGER NATURE Springer Nature is one of the world's leading global research, educational and professional publishers, created in May 2015 through the combination of Nature Publishing Group, Palgrave Macmillan, Macmillan Education and Springer Science+Business Media.

SPRINGER NATURE

Change of authorship request form - Journals (pre-acceptance)

	First name	Family name		Signature	Date
8th author					
9th author					
10th author					

Please use an additional sheet if there are more than 10 authors.

In case of author collaborations with formal agreement:

	Name of consortium/consortia	First name	Family name		Signature	Date
Representative/legal guarantor						

Both added/removed authors should complete the information in the first table under Section 6.

---- End of form ----

SPRINGER NATURE Springer Nature is one of the world's leading global research, educational and professional publishers, created in May 2015 through the combination of Nature Publishing Group, Palgrave Macmillan, Macmillan Education and Springer Science+Business Media.
